# Four new isoflavanones from *Tadehagi triquetrum*

**DOI:** 10.1007/s13659-011-0033-5

**Published:** 2011-12-16

**Authors:** Rong-Ting Zhang, Gui-Guang Cheng, Tao Feng, Xiang-Hai Cai, Xiao-Dong Luo

**Affiliations:** 1 State Key Laboratory of Phytochemistry and Plant Resources in West China, Kunming Institute of Botany, Chinese Academy of Sciences, Kunming, 650201 China; 2 Guangxi Botanical Garden of Medicinal Plants, 189 Changgang Road, Nanning, 530023 China

**Keywords:** *Tadehagi triquetrum*, isoflavanone, triquetrumone

## Abstract

Four new isoflavanones with isoprenoid units, named triquetrumones E-H (**1–4**), were isolated from the whole plants of *Tadehagi triquetrum*. The structures were elucidated on the basis of spectroscopic analyses, including application of MS, UV, IR, 1D and 2D NMR spectroscopic techniques.

**Electronic Supplementary Material:**

Supplementary material is available for this article at 10.1007/s13659-011-0033-5 and is accessible for authorized users.

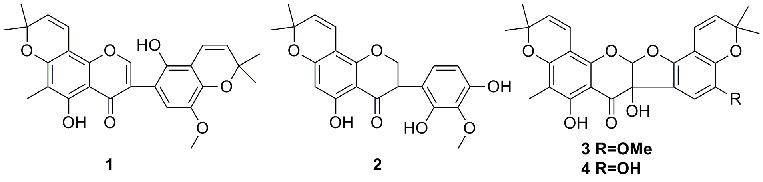

## Introduction

*Tadehagi triquetrum* (Linn.), belonging to the family of Papilionaceae, is an endemic shrub widely distributed in the southern area of Yunnan Province, China. As a traditional DAI medicine in southwest of China, it has been widely used for the treatments of anthelminthic, stomachic, antimicrobial, and inflammation. In addition, it also has been used as a nutrientand appetitive feedstuff.[1,2]

Previous phytochemical studies found a few of isoflavones and phenols from this species.[3,4] As a part of our continuing research on medicinal plants of DAI ethnopharmacy,[6–10] we investigated the chemical constituents of the whole plants of *T. triquetrum*, which led to the isolation of four new isoflavanones, named triquetrumones E–H (**1**–**4**). Isoflavanones possessing isoprenoid unit, can be considered as the characteristic constituents in this plant. This paper describes the isolation and the structural elucidation of these four new compounds.

## Results and Discussion

Compound **1** was obtained as yellow amorphous powder which showed an orange fluorescence under UV at 365 nm. The molecular formula was determined as $C_{27}H_{26}O_7$ by HRFABMS ($[M + H]^+$ at *m/z* 463.1738). The IR spectrum showed bands at 3440 (OH), 1637 (C=O), and 1604 (aromatic ring) $cm^{-1}$. In the $^1H$ NMR spectrum (Table 1), one singlet at $\delta_H$ 8.03 (s, H-2), an aromatic C-methyl group at $\delta_H$ 2.12 (s, H-11), two phenolic hydroxyl at $\delta_H$ 12.62 (br. s) and 8.05 (br. s), one methoxyl group at $\delta_H$ 3.84 (s) and two 2,2-dimethylpyran rings at $\delta_H$ 5.62 (d, $J$ = 9.9, H-5''), 6.71 (d, $J$ = 9.9, H-4''), 1.48 (s, H-7'',8'') and 5.63 (d, $J$ = 9.9, H-5'''), 6.82 (d, $J$ = 9.9, H-4'''), 1.50 (s, Me-7''' and Me-8''') were observed. In addition, the $^{13}C$ NMR spectrum (Table 2) displayed 15 skeletal C atoms: one carbonyl at $\delta_C$ 181.9, 13 quaternary C-atoms at $\delta_C$ 104.7–159.1, and as well one CH group at $\delta_C$ 154.7. Considering the above NMR data and the orange fluorescence under UV at 365nm, the structure was proposed to be an isoflavone, possessing two isoprenoids units.[3]

The HMBC correlations (Fig. 1) of H-5''/C-8 and H-4'' with C-6'', C-7, and C-9, and of H-5'''/C-3', and H-4''' with C-6''' and C-4' suggested that two dimethylpyrano rings were condensed to C-7/8 and C-4'/3', respectively. A methyl at $\delta_H$ 2.12 was assigned to connection with C-6 by its correlations with C-5, 6, 7 in the HMBC spectrum. Moreover, two OH groups were placed at C-5 and C-2', respectively, and a methoxyl group was positioned at C-5' by HMBC experiments. Complete analysis of the $^1H$ and $^{13}C$ NMR, HSQC, HMBC data established the structure of **1** as (5,2'-dihydroxy-5'-methoxyl-6-methyl[6'',6''-dimethylpyrano-(2'',3'':7,8)]-[6''',6'''-dimethyl-pyrano(2''',3''':4',3')]-isoflavanone, and named triquetrumone E.

**1** **2** **3** R=OMe **4** R=OH

Compound **2** was isolated as yellow amorphous powder. It gave a molecular formula $C_{21}H_{20}O_7$ by HRFABMS ($[M + H]^+$ at *m/z* 385.1274). Bands for OH and carbonyl functional groups were assumed by IR absorption at 3421 and 1647 and UV absorptions at 270 nm were typical for an isoflavanone.[11] The NMR spectra of compound **2** (Tables 1 and 2) were similar to those of 1''',2'''-dehydrocyclokievitone,[12] except for the appearance of a methyoxyl group ($\delta_H$ 3.78, $\delta_C$ 60.7) in **2**, which was also supported by its molecular formula. The

*To whom correspondence should be addressed. E-mail: xdluo@mail.kib.ac.cn

**Table 1.** **$^{1}$H NMR data of 1–4.**

| position | **1**[a] | **2**[b] | **3**[c] | **4**[b] |
|---|---|---|---|---|
| 2 | 8.03, s | 4.53, dd (5.5, 10.9); 4.64, t (10.9) | 6.32, s | 6.29, s |
| 3 | | 4.27, dd (10.8, 5.5) | | |
| 6 | | 5.88, s | | |
| 11 | 2.12, s | | 1.92, s | 1.92, s |
| 5' | | 6.38, d (8.4) | | |
| 6' | 6.57 , s | 6.69, d (8.4) | 6.89, s | 6.78, s |
| 4" | 6.71, d (9.9) | 6.51, d (10.1) | 6.46, d (9.9) | 6.57, d (10.0) |
| 5" | 5.62, d (9.9) | 5.60, d (10.1) | 5.77, d (9.9) | 5.67, d (10.0) |
| 7" | 1.48, s | 1.41, s | 1.38, s | 1.38, s |
| 8" | 1.48, s | 1.42, s | 1.42, s | 1.41, s |
| 4''' | 6.82, d (9.9) | | 6.57, d (10.0) | 6.46, d (9.9) |
| 5''' | 5.63, d (9.9) | | 5.68, d (10.0) | 5.76, d (9.9) |
| 7''' | 1.50, s | | 1.41, s | 1.38, s |
| 8''' | 1.50, s | | 1.45, s | 1.41, s |
| OMe | 3.84, s | 3.78, s | 3.73, s | |
| OH(5) | 12.62, br. s | 12.44, br. s | 12.27, br. s | 12.27, br. s |
| OH(2') | 8.05, br. s | 8.15, br. s | | |
| OH(4') | | 8.30, br. s | | |
| OH(5') | | | | 7.50, br. s |
| OH(3) | | | 6.08, br. s | 6.05, br. s |

[a]Measured in $CDCl_3$ at 400MHz; [b]Measured in acetone-$d_6$ at 500MHz; [c]Measured in acetone-$d_6$ at 400MHz.

methyoxyl group was paced at C-3', supported by the HMBC correlations (Fig. 1). Thus, compound **2** was established to be 5,2',4'-trihydroxy-3'-methoxy[6",6"-dimethylpyrano(2",3":7,8)]-isoflavavone, and named triquetrumone F.

Compound **3** appeared as yellow amorphous powder whose molecular was established as $C_{27}H_{26}O_8$ by HRFABMS at *m/z* 479.1723 (calcd. for $C_{27}H_{27}O_8$, 479.1706), which indicated 15 degrees of unsaturation. *T. triquetrum* are known to contain three 5-hydroxy-coumaronochromones, and all of which fluoresce on silica gel thin-layer plates under 365 nm of UV light.[3] Take UV, MS, and NMR data into account, compound **3** can be most logically formulated as an isoflavone, in which C-2 and C-2' was linked by an ether oxygen to form a pentacyclic ring system.[13] The $^{1}$H NMR spectrum (Table 1) of **3** revealed two 2,2-dimethylpyrane rings at $\delta_H$ 5.77 (d, $J$ = 9.9, H-5"), 6.46 (d, $J$ = 9.9, H-4"), 1.38 (s, Me-7") and 1.42 (s, Me-8"), together with 5.68 (d, $J$ = 10.0, H-5'''), 6.57 (d, $J$ = 10.0, H-4'''), 1.41 (s, Me-7''') and 1.45 (s, Me-8'''), associated with an aromatic C-methyl group at $\delta_H$1.92 (s) and two singlets at $\delta_H$ 6.32 (s, H-2) and 6.89 (s, H-6'), respectively.

The $^{13}$C and $^{1}$H NMR spectra of **3** implied that this compound might be an analogue of triquetrumone B.[3] Careful comparison of the NMR data of two compounds revealed that one more OH group might be appeared at C-3, suggested by downshift signals at $\delta_C$ 79.4 (s, C-3), $\delta_H$ 6.08 (br. s, OH) in **3**. The proposal was further supported by the HMBC correlations (Fig. 1) between OH-3 with $\delta_C$ 193.4 (s, C-4), 111.5 (d, C-2) and 79.4 (s, C-3). Thus, **3** was established as 3,5-dihydroxy-5'-methoxyl-6-methyl[6",6"-dimethylpyrano(2",3":7,8)]-[6''',6'''-dimethylpyrano(2''',3''':2',3')]coumaronochromone, and named triquetrumone G.

Compound **4** was obtained as yellow amorphous powder. The HRFABMS clearly showed the $[M + H]^+$ in agreement with the molecular formula $C_{26}H_{24}O_8$. The UV, IR, and NMR spectroscopic data also suggested a coumaronochromone structure for **4**,[14] which was very similar to those of **3**. Detailed comparison of the NMR data of two compounds revealed that a hydroxyl group was appeared on ring C of **4**, instead of the methyoxyl in **3**, concurring with its molecular formula. The assignment was also supported by the HMBC correlations (Fig. 1) of $\delta_H$ 7.50 (*s*, OH-5') with $\delta_C$ 106.9 (s, C-3'), 142.1 (s, C-4') and 143.0 (s, C-5'). Thus, the structure of **4** was established as 3,5,5'-trihydroxy-6-methyl[6",6"-dimethylpyrano(2",3":7,8)]-[6''',6'''-dimethypyrano(2''',3''':2',3')]coumaronochromone, and named triquetrumone H.

**Fig 1.** Key HMBC correlations of **1**–**4**.

## Experimental Section

**General Experimental Procedures**. Column chromatography (CC): Silica gel (200–300 mesh, *Qingdao Marine Chemical corporation*, China) and Sephadex LH-20 (*Amersham Biosciences,* Sweden); TLC monitoring, visualization by heating the silica gel plates sprayed with 10% $H_2SO_4$ in EtOH. Optical rotations: *Horiba SEPA-300* polarimeter. UV spectra: *Shimadzu 210-A* double-beam spectrophotometer; $\lambda_{max}$ (logε) in nm. IR Spectra: *Brucker Tensor 27* spectrometer ; KBr pellets; in $cm^{-1}$. NMR Spectra: *Bruker AM-500* spectrometers; $\delta$ in ppm with $SiMe_4$ as internal standard, $J$ in Hz. MS: *VG Autospec-3000* and *API QSTAR-Palsar-I* spectrometer.

**Plant Material.** The whole plants of *Tadehagi triquetrum* were collected from Xishuangbanna, Yunnan Province, China, in April 2007. Its identity was confirmed by Mr. Jing-Yun Cui, and a voucher specimen (NO. 2007042) has been deposited in the State Key Laboratory of Phytochemistry and Plant Resources in West China, Kunming Institute of Botany, Chinese Academy of Sciences.

**Extraction and Isolation.** The dried and milled sample (10 kg) were soaked in 95 % EtOH (40 L × 3) under reflux (48 h × 3), and the solvent was evaporated *in vacuo*. The

**Table 2.** $^{13}C$ **NMR data of 1–4.**

| pos. | 1[a] | 2[b] | 3[c] | 4[b] |
|---|---|---|---|---|
| 2 | 154.7, CH | 71.2, $CH_2$ | 111.5, CH | 111.4, CH |
| 3 | 123.4, C | 47.5, CH | 79.4, C | 79.3, C |
| 4 | 181.9, C | 198.5, C | 193.4, C | 193.6, C |
| 5 | 159.1, C | 165.0, C | 162.4, C | 162.3, C |
| 6 | 109.8, C | 97.6, CH | 107.0, C | 107.1, C |
| 7 | 158.3, C | 162.4, C | 161.4, C | 161.3, C |
| 8 | 100.5, C | 102.3, C | 102.6, C | 102.5, C |
| 9 | 150.1, C | 158.2, C | 152.1, C | 152.7, C |
| 10 | 104.7, C | 103.9, C | 100.7, C | 101.6, C |
| 11 | 7.3, C | | 6.8, $CH_3$ | 6.8, C |
| 1' | 110.8, C | 114.0, C | 117.5, C | 118.2, C |
| 2' | 146.1, C | 149.6, C | 150.3, C | 148.8, C |
| 3' | 113.6, C | 136.4, C | 107.6, C | 106.9, C |
| 4' | 144.5, C | 150.9, C | 145.4, C | 142.1, C |
| 5' | 142.8, C | 108.3, CH | 146.0, C | 143.0, C |
| 6' | 114.1, CH | 125.7, CH | 110.6, CH | 111.7, CH |
| 4'' | 114.6, CH | 116.0, CH | 115.8, CH | 115.8, CH |
| 5'' | 129.4, CH | 127.3, CH | 128.1, CH | 128.0, CH |
| 6'' | 78.3, C | 78.7, C | 79.4, C | 79.3, C |
| 7'' | 28.3, $CH_3$ | 28.3, $CH_3$ | 28.0, $CH_3$ | 27.7, $CH_3$ |
| 8'' | 28.3, $CH_3$ | 28.5, $CH_3$ | 28.7, $CH_3$ | 27.8, $CH_3$ |
| 4''' | 117.8, CH | | 116.3, CH | 116.3, CH |
| 5''' | 127.5, CH | | 131.6, CH | 131.7, CH |
| 6''' | 76.5, CH | | 77.6, C | 77.8, C |
| 7''' | 27.8, $CH_3$ | | 28.6, $CH_3$ | 28.5, $CH_3$ |
| 8''' | 27.8, $CH_3$ | | 29.3, $CH_3$ | 28.6, $CH_3$ |
| OMe | 57.4, $CH_3$ | 60.7, $CH_3$ | 57.5, $CH_3$ | |

[a]Recorded in $CDCl_3$ at 100MHz; [b]Recorded in acetone-$d_6$ at 100MHz; [c]Recorded in acetone-$d_6$ at 125 MHz.

residue was partitioned between EtOAc and $H_2O$. The EtOAc extract (80 g) was subjected to CC ($CHCl_3/Me_2CO$ 10:1→1:1): eight fractions (*Fr.1–8*) by TLC. *Fr.3* (15 g) was repeatedly subjected to CC (1. Silica-G petroleum ether/$Me_2CO$ 10:1→2:1; 2. *Sephadex* LH-20, $CHCl_3$/MeOH 1:1) to afford **1** (6 mg). *Fr.4* (12 g) was purified by repeated CC ($SiO_2$, petrolrum ether/$Me_2CO$ 3:1) to afford **2** (21 mg). *Fr.5* (10 g) was resubmitted to CC (1. $SiO_2$, $CHCl_3$/MeOH 70:1→50:1; 2. *Sephadex* LH-20, MeOH/$H_2O$ 9:1) to provide **3** (5 mg) and **4** (25 mg).

**Triquetrumone E (1):** yellow amorphous powder, UV (MeOH) $\lambda_{max}$ (log ε) 273 (4.49), 212 (4.51) nm. IR (KBr) $\nu_{max}$ 3440, 2924, 1637, 1604 $cm^{-1}$. $^1H$ and $^{13}C$ NMR data see Tables 1 and 2. ESIMS (positive): 463 ($[M + H]^+$). HRFABMS (positive): 463.1738 ($[M + H]^+$, $C_{27}H_{27}O_7$, calcd. 463.1757).

**Triquetrumone F (2):** yellow amorphous powder, $[\alpha]^{20}_{D}$ −1.63 (*c* 0.002, $Me_2CO$). UV (MeOH) $\lambda_{max}$ (log ε) 270 (4.24), 207 (4.42) nm. IR (KBr) $\nu_{max}$ 3421, 1647, 1598 $cm^{-1}$. $^1H$ and $^{13}C$ NMR data see Tables 1 and 2. ESIMS (positive): 385 ($[M + H]^+$). HRFABMS (positive): 385.1274 ($[M + H]^+$, $C_{21}H_{21}O_7$, calcd. 385.1287).

**Triquetrumone G (3):** yellow amorphous powder, $[\alpha]^{20}_{D}$ −44.5 (*c* 0.001, $Me_2CO$). UV(MeOH) $\lambda_{max}$ (log ε) 273 (4.01), 268 (4.02), 212 (4.39) nm. IR (KBr) $\nu_{max}$ 3441, 1632, 1600 $cm^{-1}$. $^1H$ and $^{13}C$-NMR data see Tables 1 and 2. EIMS: 478 $[M]^+$. HRFABMS (positive): 479.1723 ($[M + H]^+$, $C_{27}H_{27}O_8$, calcd. 479.1706).

**Triquetrumone H (4):** yellow amorphous powder, $[\alpha]^{20}_{D}$ −32.3 (*c* 0.001, $Me_2CO$). UV (MeOH) $\lambda_{max}$ (log ε) 274 (4.34), 268 (4.34), 212 (4.45) nm. IR (KBr) $\nu_{max}$ 3424, 1635, 1599 $cm^{-1}$. $^1H$ and $^{13}C$ NMR data see Tables 1 and 2. EIMS: 464 $[M]^+$. HRFABMS (positive): 465.1556 ($[M + H]^+$, $C_{26}H_{25}O_8$, calcd. 465.1549).

## Electronic Supplementary Material

Supplementary material is available in the online version of this article at http://dx.doi.org/10.1007/s13659-011-0033-5 and is accessible for authorized users.

## Acknowledgments

The authors are grateful to National Basic Research Program of China (973 Program 2009CB522300)，and the Chinese Academy of Science (KSCX2-EW-R-15) for financial support and to the members of the analytical group in the State Key Laboratory of Phytochemistry and Plant Resources in West China, Kunming Institute of Botany, Chinese Academy of Sciences, for the spectral measurements.

**Open Access** This article is distributed under the terms of the Creative Commons Attribution License which permits any use, distribution, and reproduction in any medium, provided the original author(s) and source are credited.

## Electronic supplementary material

Supplementary material, approximately 1.30 MB.
