# Supplementary material for: Four new isoflavanones from Tadehagi triquetrum
Source: Nat Prod Bioprospect. 2011 Dec 16;1(3):121–3. doi: 10.1007/s13659-011-0033-5 (PMC4131644; doi:10.1007/s13659-011-0033-5)
Supplement: Supplementary file 1 — Supplementary material, approximately 1.30 MB. [file 13659_2011_33_MOESM1_ESM.pdf]

Electronic Supplementary Material

Four new isoflavanones from *Tadehagi triquetrum*

Rong-Ting ZHANG,[a,b] Gui-Guang CHENG,[a] Tao FENG,[a] Xiang-Hai CAI,[a] and Xiao-Dong LUO[a,*]

[a]State Key Laboratory of Phytochemistry and Plant Resources in West China, Kunming Institute of Botany, Chinese Academy of Sciences, Kunming 650201, China

[b]Guangxi Botanical Garden of Medicinal Plants, 189 Changgang Road, Nanning 530023, China

Received 19 November 2011; Accepted 8 December 2011

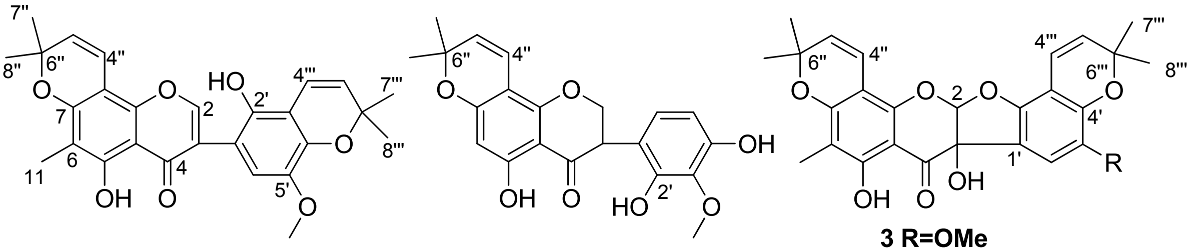

1 **2** **3** R=OMe **4** R=OH

Structures of compounds **1**–**4**

*To whom correspondence should be addressed. E-mail: xdluo@mail.kib.ac.cn

wtt3 H

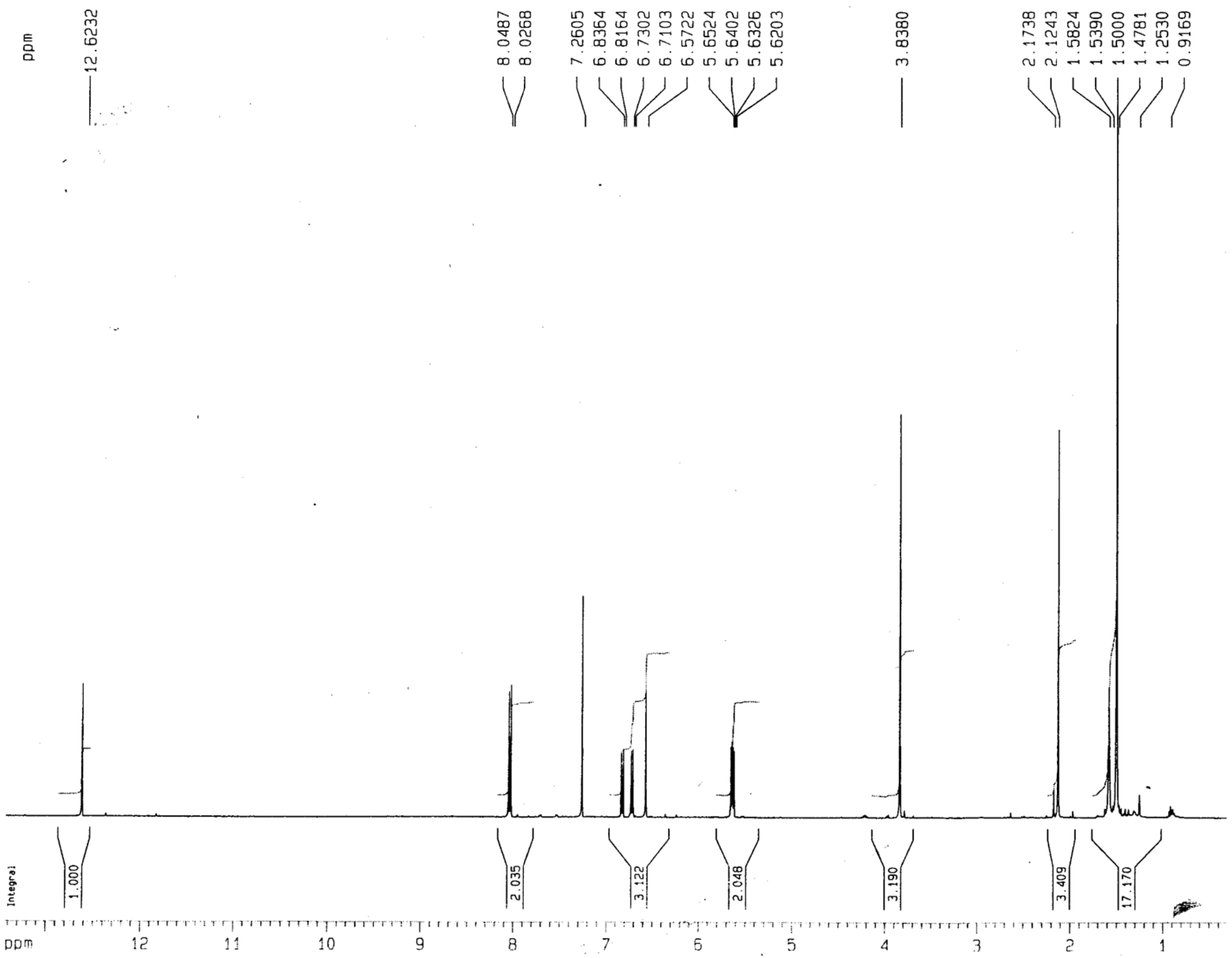

Current Data Parameters

NAME	wtt3
EXPNO	21
PROCNO	1

F2 - Acquisition Parameters

Date_	20080403
Time	9.16
INSTRUM	spect
PROBHD	5 mm DUL 13C-1
PULPROG	zg
TD	32768
SOLVENT	CDCl3
NS	1
DS	0
SWH	8992.806 Hz
FIDRES	0.274439 Hz
AQ	1.8219508 sec
RG	256
DW	55.600 usec
DE	6.00 usec
TE	292.2 K
D1	1.00000000 sec
MCREST	0.00000000 sec
MCWRK	0.01500000 sec

======== CHANNEL f1 ========

NUC1	1H
P1	10.00 usec
PL1	-4.00 dB
SFO1	500.0325001 MHz

F2 - Processing parameters

SI	16384
SF	500.0300110 MHz
WDW	GM
SSB	0
LB	-0.40 Hz
GB	0.2
PC	1.00

1D NMR plot parameters

CX	22.00 cm
CY	23.00 cm
F1P	13.429 ppm
F1	6715.09 Hz
F2P	0.313 ppm
F2	156.43 Hz
PPMCM	0.59621 ppm/cm
HZCM	298.12103 Hz/cm

wtt3 c13

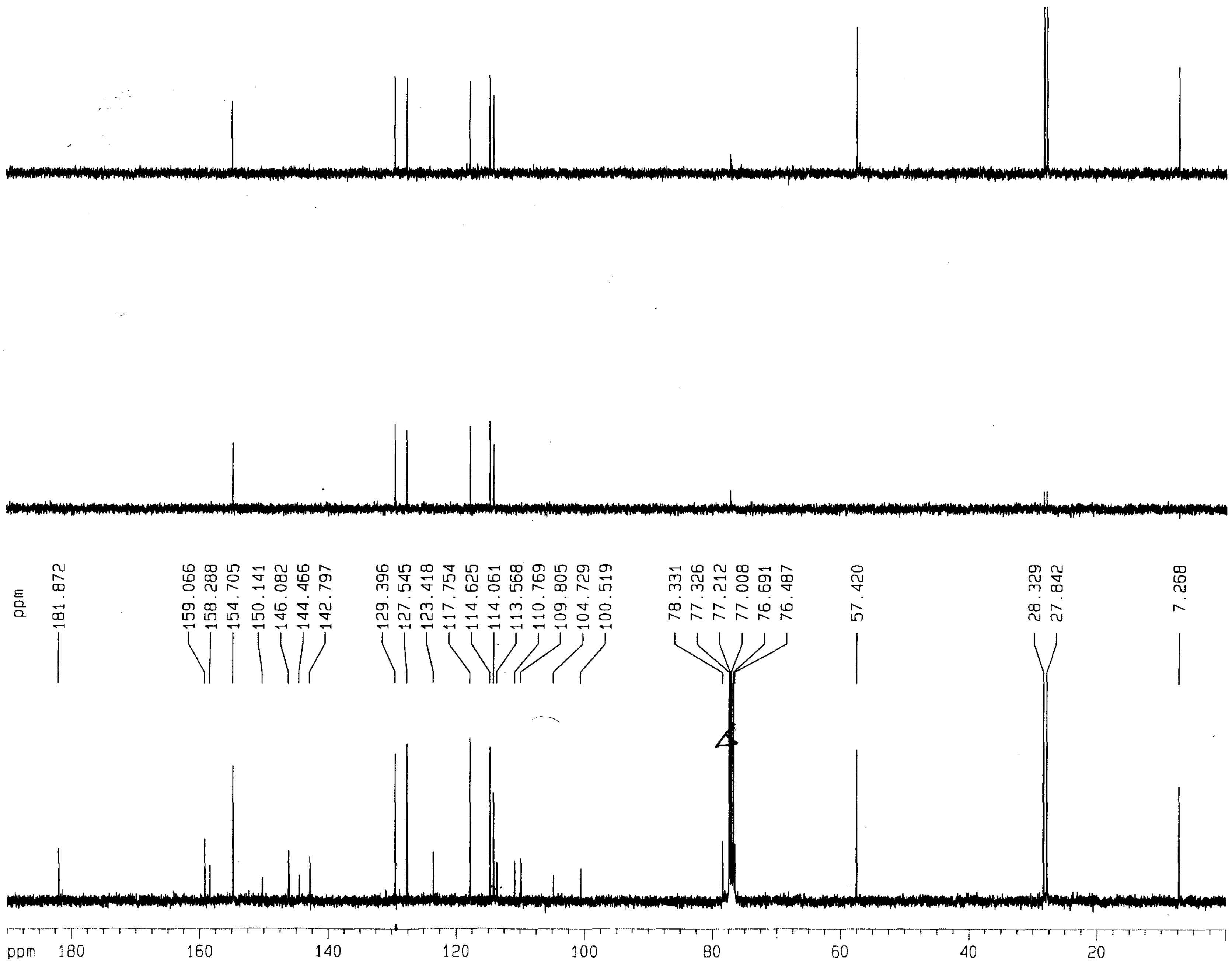

Current Data Parameters

NAME	wtt3
EXPNO	2
PROCNO	1

F2 - Acquisition Parameters

Date_	20080410
Time	5.09
INSTRUM	av400
PROBHD	5 mm QNP 1H/15
PULPROG	zgdc
TD	32768
SOLVENT	CDCl3
NS	2680
DS	2
SWH	23584.906 Hz
FIDRES	0.719754 Hz
AQ	0.6947528 sec
RG	45.3
DW	21.200 usec
DE	6.00 usec
TE	291.6 K
D1	3.00000000 sec
d11	0.03000000 sec
MCREST	0.00000000 sec
MCWRK	0.01500000 sec

======== CHANNEL f1 ========

NUC1	13C
P1	9.40 usec
PL1	-4.00 dB
SFO1	100.6239976 MHz

======== CHANNEL f2 ========

CPDPRG2	waltz16
NUC2	1H
PCPD2	90.00 usec
PL2	-3.00 dB
PL12	14.00 dB
SFO2	400.1316005 MHz

F2 - Processing parameters

SI	32768
SF	100.6127720 MHz
WDW	EM
SSB	0
LB	1.00 Hz
GB	0
PC	1.50

1D NMR plot parameters

CX	22.00 cm
CY	6.00 cm
F1P	190.000 ppm
F1	19116.43 Hz
F2P	-0.000 ppm
F2	-0.00 Hz
PPMCM	8.63636 ppm/cm
HZCM	868.92847 Hz/cm

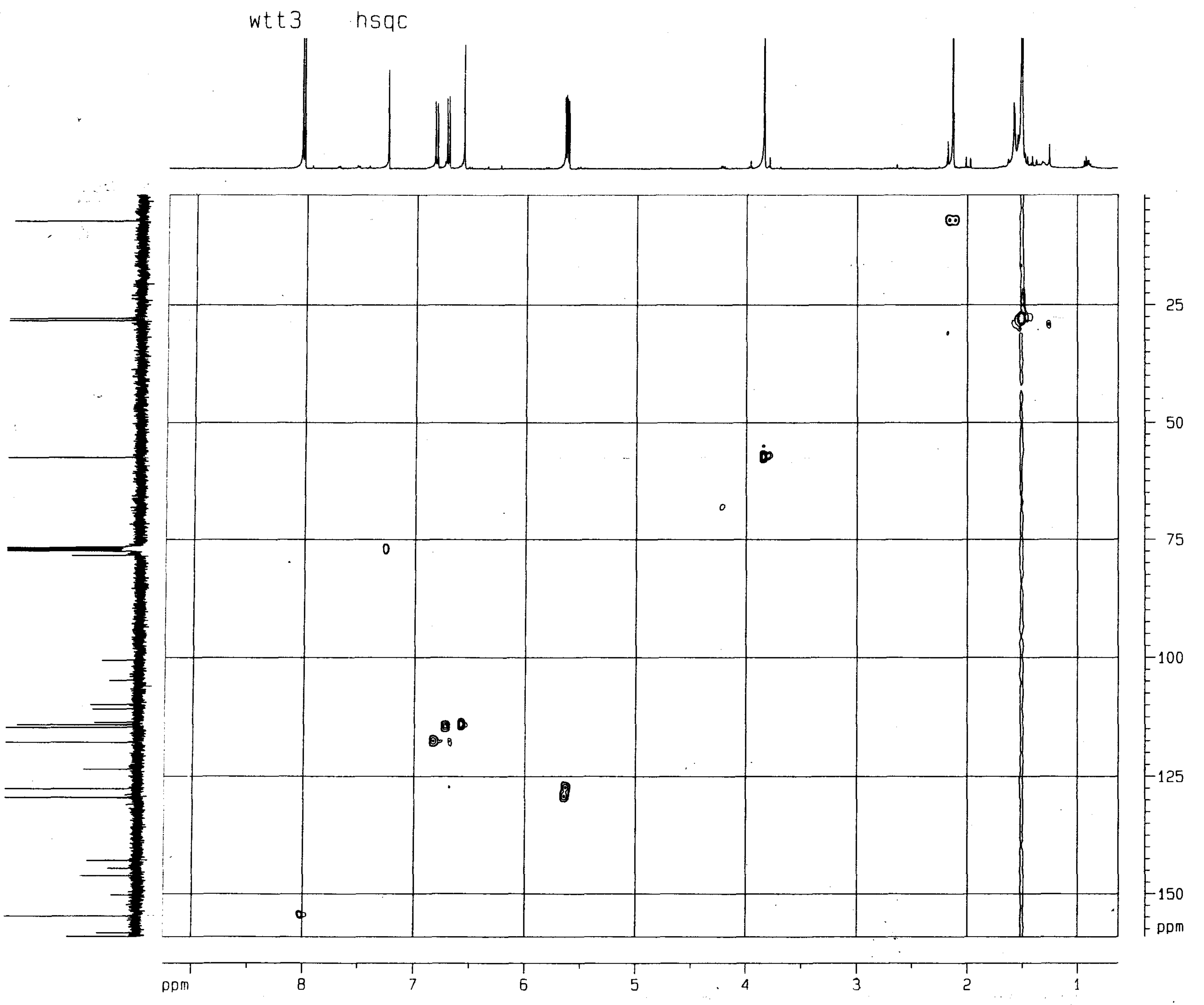

Current Data Parameters

NAME	wtt3
EXPNO	26
PROCNO	1

F2 - Acquisition Parameters

Date_	20080411
Time	15.55
INSTRUM	spect
PROBHD	5 mm BBI 1H-BB
PULPROG	hsqcetgpsi2
TD	1024
SOLVENT	CDCl3
NS	4
DS	16
SWH	6510.417 Hz
FIDRES	6.357829 Hz
AQ	0.0786832 sec
RG	11585.2
DW	76.800 usec
DE	6.00 usec
TE	0.0 K
CNST2	145.0000000
d0	0.00000300 sec
D1	1.50000000 sec
d4	0.00172414 sec
d11	0.03000000 sec
d13	0.00000400 sec
D16	0.00020000 sec
D24	0.00086207 sec
DELTA	0.00127440 sec
DELTA1	0.00120800 sec
DELTA2	0.00006207 sec
DELTA3	0.00052414 sec
IN0	0.00002368 sec
MCREST	0.00000000 sec
MCWRK	0.25000051 sec
ST1CNT	64

======== CHANNEL f1 ========

NUC1	1H
P1	9.20 usec
p2	18.40 usec
P28	2000.00 usec
PL1	-1.00 dB
SFO1	500.0333502 MHz

======== CHANNEL f2 ========

CPDPRG2	garp
NUC2	13C
P3	12.00 usec
p4	24.00 usec
PCPD2	75.00 usec
PL2	-1.00 dB
PL12	15.50 dB
SFO2	125.7427026 MHz

====== GRADIENT CHANNEL =====

GPNAM1	SINE.100
GPNAM2	SINE.100
GPNAM3	SINE.100
GPNAM4	SINE.100
GPX1	0.00 %
GPX2	0.00 %
GPX3	0.00 %
GPX4	0.00 %
GPY1	0.00 %
GPY2	0.00 %
GPY3	0.00 %
GPY4	0.00 %
GPZ1	80.00 %
GPZ2	20.10 %
GPZ3	11.00 %
GPZ4	-5.00 %
P16	1000.00 usec
P19	600.00 usec

F1 - Acquisition parameters

ND0	2
TD	128
SFO1	125.7425 MHz
FIDRES	164.994720 Hz
SW	167.957 ppm
FnMODE	Echo-Antiecho

F2 - Processing parameters

SI	1024
SF	500.0300069 MHz
WDW	QSINE
SSB	2
LB	0.00 Hz
GB	0
PC	1.40

F1 - Processing parameters

SI	1024
MC2	echo-antiecho
SF	125.7324203 MHz
WDW	QSINE
SSB	2
LB	0.00 Hz
GB	0

2D NMR plot parameters

CX2	18.00 cm
CX1	14.00 cm
F2PLO	9.256 ppm
F2LO	4627.61 Hz
F2PHI	0.634 ppm
F2HI	317.01 Hz
F1PLO	159.172 ppm
F1LO	20013.04 Hz
F1PHI	1.699 ppm
F1HI	213.67 Hz
F2PPMCM	0.47893 ppm/cm
F2HZCM	239.47821 Hz/cm
F1PPMCM	11.24802 ppm/cm
F1HZCM	1414.24048 Hz/cm

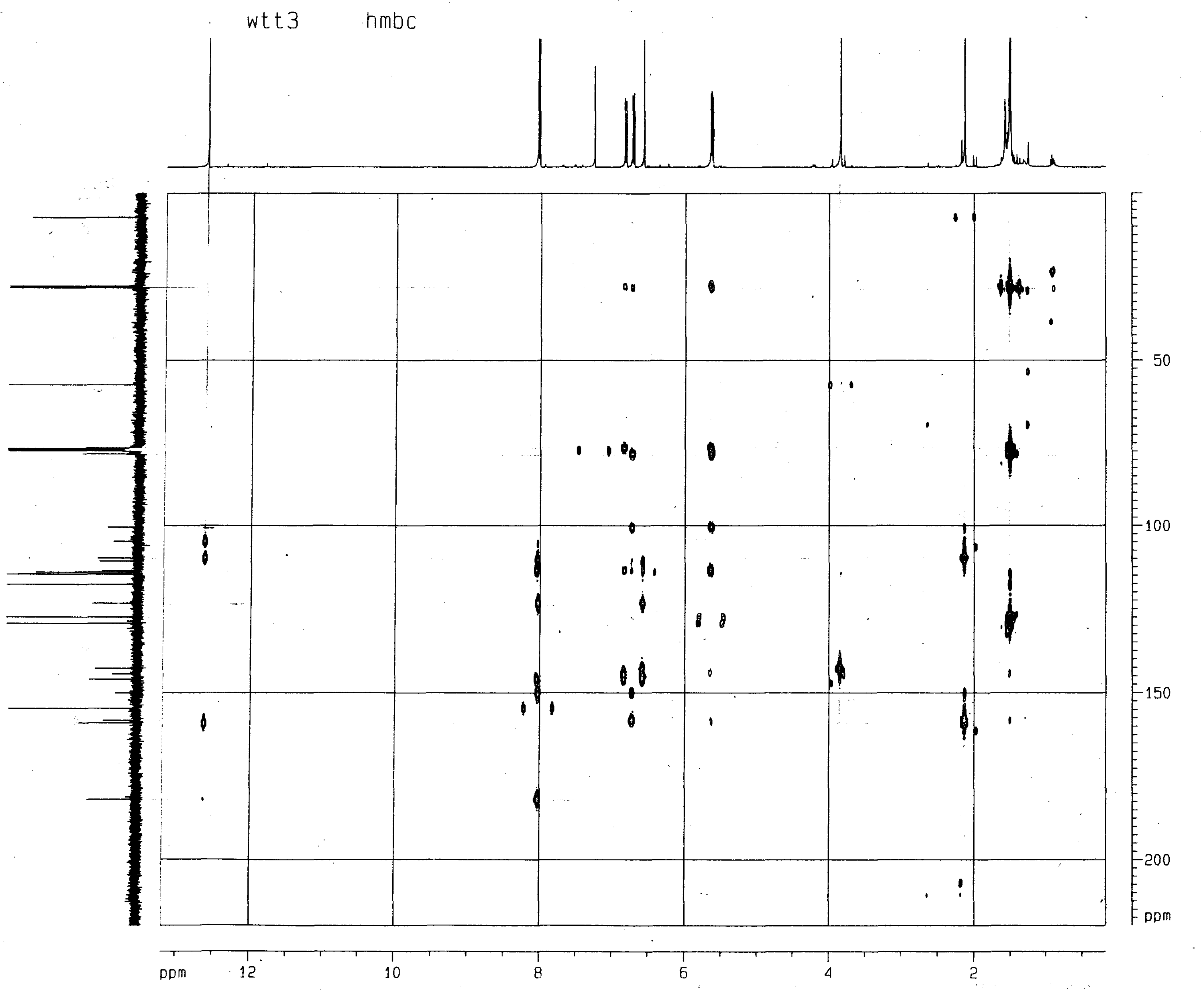

Current Data Parameters

NAME	wtt3
EXPNO	27
PROCNO	1

F2 - Acquisition Parameters

Date_	20080411
Time	16.10
INSTRUM	spect
PROBHD	5 mm BBI 1H-BB
PULPROG	inv4gplplrndqf
TD	2048
SOLVENT	Acetone
NS	32
DS	16
SWH	6510.417 Hz
FIDRES	3.178914 Hz
AQ	0.1573364 sec
RG	23170.5
DW	76.800 usec
DE	6.00 usec
TE	0.0 K
CNST2	145.0000000
d0	0.00000300 sec
D1	1.39999998 sec
d2	0.00344828 sec
D6	0.08340000 sec
d13	0.00000400 sec
D16	0.00020000 sec
IN0	0.00001807 sec
MCREST	0.00000000 sec
MCWRK	1.39999998 sec

======== CHANNEL f1 ========

NUC1	1H
P1	9.20 usec
p2	18.40 usec
PL1	-1.00 dB
SFO1	500.0333502 MHz

======== CHANNEL f2 ========

NUC2	13C
P3	12.00 usec
PL2	-1.00 dB
SFO2	125.7464746 MHz

====== GRADIENT CHANNEL =====

GPNAM1	SINE.100
GPNAM2	SINE.100
GPNAM3	SINE.100
GPX1	0.00 %
GPX2	0.00 %
GPX3	0.00 %
GPY1	0.00 %
GPY2	0.00 %
GPY3	0.00 %
GPZ1	50.00 %
GPZ2	30.00 %
GPZ3	40.10 %
P16	1000.00 usec

F1 - Acquisition parameters

ND0	2
TD	128
SFO1	125.7467 MHz
FIDRES	216.113419 Hz
SW	219.986 ppm
FnMODE	QF

F2 - Processing parameters

SI	1024
SF	500.0300078 MHz
WDW	SINE
SSB	0
LB	0.00 Hz
GB	0
PC	1.40

F1 - Processing parameters

SI	512
MC2	QF
SF	125.7329049 MHz
WDW	SINE
SSB	0
LB	0.00 Hz
GB	0

2D NMR plot parameters

CX2	18.00 cm
CX1	14.00 cm
F2PLO	13.195 ppm
F2LO	6597.65 Hz
F2PHI	0.174 ppm
F2HI	87.23 Hz
F1PLO	219.930 ppm
F1LO	27652.43 Hz
F1PHI	-0.080 ppm
F1HI	-10.09 Hz
F2PPMCM	0.72334 ppm/cm
F2HZCM	361.68979 Hz/cm
F1PPMCM	15.71501 ppm/cm
[illegible]	[illegible]75.89417 Hz/cm

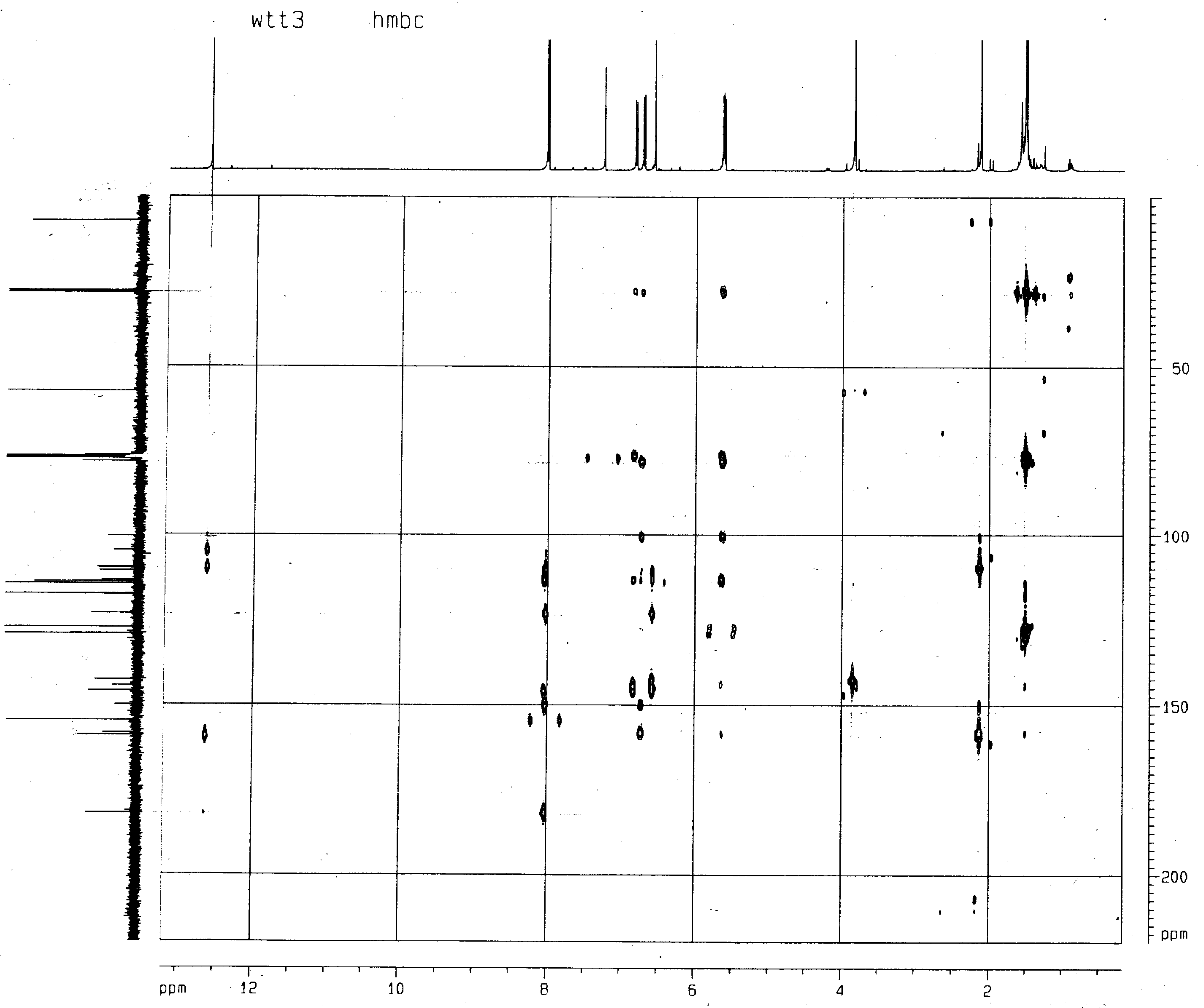

Current Data Parameters

NAME	wtt3
EXPNO	27
PROCNO	1

F2 - Acquisition Parameters

Date_	20080411
Time	16.10
INSTRUM	spect
PROBHD	5 mm BBI 1H-BB
PULPROG	inv4gplplrndqf
TD	2048
SOLVENT	Acetone
NS	32
DS	16
SWH	6510.417 Hz
FIDRES	3.178914 Hz
AQ	0.1573364 sec
RG	23170.5
DW	76.800 usec
DE	6.00 usec
TE	0.0 K
CNST2	145.0000000
d0	0.00000300 sec
D1	1.39999998 sec
d2	0.00344828 sec
D6	0.08340000 sec
d13	0.00000400 sec
D16	0.00020000 sec
IN0	0.00001807 sec
MCREST	0.00000000 sec
MCWRK	1.39999998 sec

======== CHANNEL f1 ========

NUC1	1H
P1	9.20 usec
p2	18.40 usec
PL1	-1.00 dB
SFO1	500.0333502 MHz

======== CHANNEL f2 ========

NUC2	13C
P3	12.00 usec
PL2	-1.00 dB
SFO2	125.7464746 MHz

====== GRADIENT CHANNEL =====

GPNAM1	SINE.100
GPNAM2	SINE.100
GPNAM3	SINE.100
GPX1	0.00 %
GPX2	0.00 %
GPX3	0.00 %
GPY1	0.00 %
GPY2	0.00 %
GPY3	0.00 %
GPZ1	50.00 %
GPZ2	30.00 %
GPZ3	40.10 %
P16	1000.00 usec

F1 - Acquisition parameters

ND0	2
TD	128
SFO1	125.7467 MHz
FIDRES	216.113419 Hz
SW	219.986 ppm
FnMODE	QF

F2 - Processing parameters

SI	1024
SF	500.0300078 MHz
WDW	SINE
SSB	0
LB	0.00 Hz
GB	0
PC	1.40

F1 - Processing parameters

SI	512
MC2	QF
SF	125.7329049 MHz
WDW	SINE
SSB	0
LB	0.00 Hz
GB	0

2D NMR plot parameters

CX2	18.00 cm
CX1	14.00 cm
F2PLO	13.195 ppm
F2LO	6597.65 Hz
F2PHI	0.174 ppm
F2HI	87.23 Hz
F1PLO	219.930 ppm
F1LO	27652.43 Hz
F1PHI	-0.080 ppm
F1HI	-10.09 Hz
F2PPMCM	0.72334 ppm/cm
F2HZCM	361.68979 Hz/cm
F1PPMCM	15.71501 ppm/cm
F1HZCM	1975.89417 Hz/cm

D:\LUODX\2007-06-06\WTT3 2008-04-10 23:41:36
MeOH (95):Water((5)0.5% formic acid)

WTT3 #29-31 RT: 0.39-0.42 AV: 3 SB: 6 0.13-0.20 NL: 9.28E5
T: + c ms [150.00-800.00]

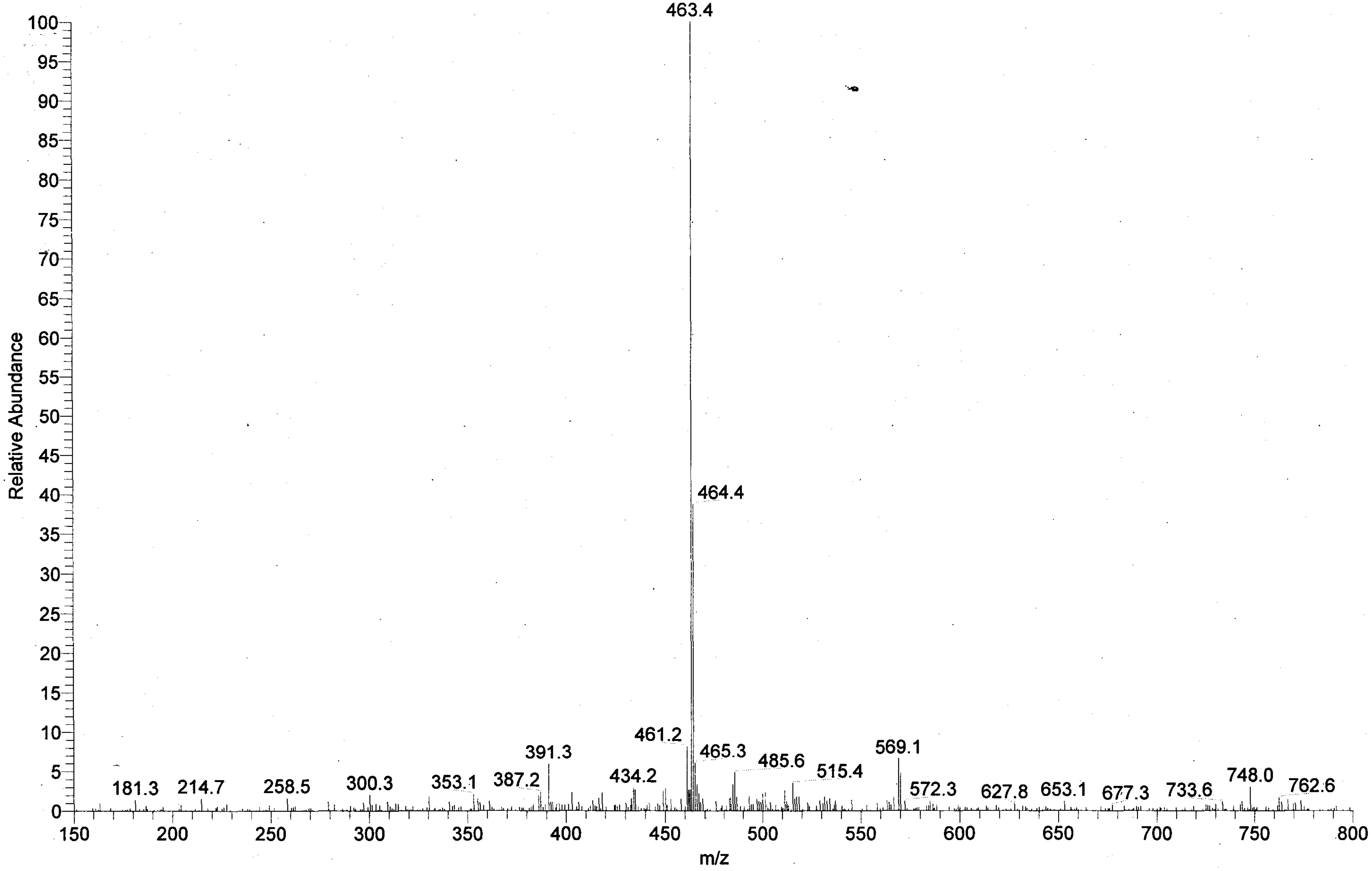

File:0106FA4 Ident:7 SMO(1,5) PKD(5,3,5,0.50%,0.0,0.00%,F,F) SPEC(Heights,Centroid) Acq: 6-JAN-»
AutoSpec FAB+ Voltage BpI:335308 TIC:19193168 Flags:NORM
File Text:Res5000 Gly Wtt-3

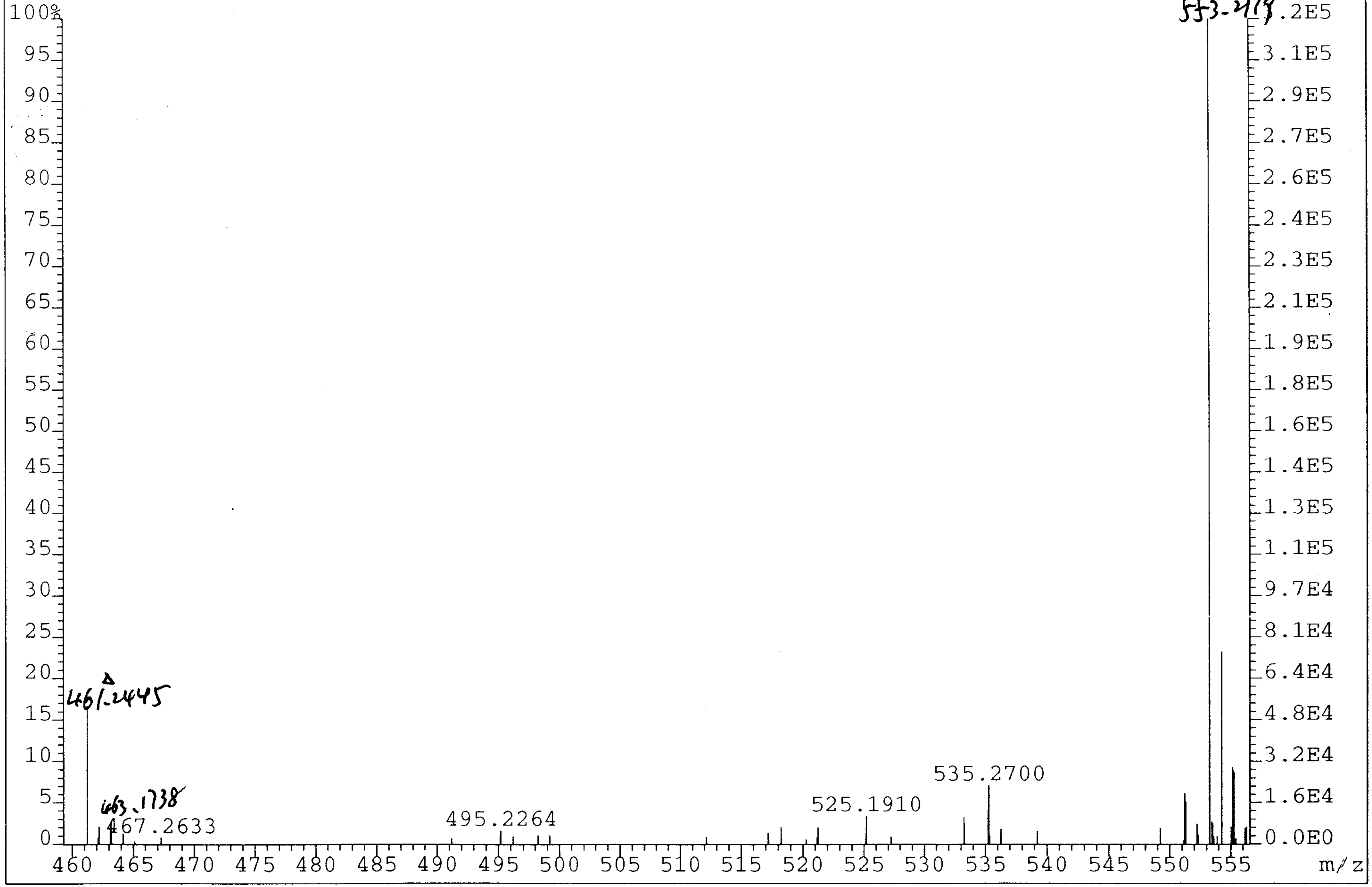

File:0106FA4 Ident:7 SMO(1,5) PKD(5,3,5,0.50%,0.0,0.00%,F,F)
AutoSpec FAB+ Voltage BpI:335308 TIC:19193168 Flags:NORM
File Text:Res5000 Gly Wtt-3
Heteroatom Max: 60 Ion: Both Even and Odd
Limits:

				-0.5	0	0	0
463.173794	10.0			20.0	200	400	10
**Mass**	**mDa**	**PPM**	**Calc. Mass**	**DBE**	**C**	**H**	**O**
463.173794	1.9	4.1	463.175679	14.5	27	27	7

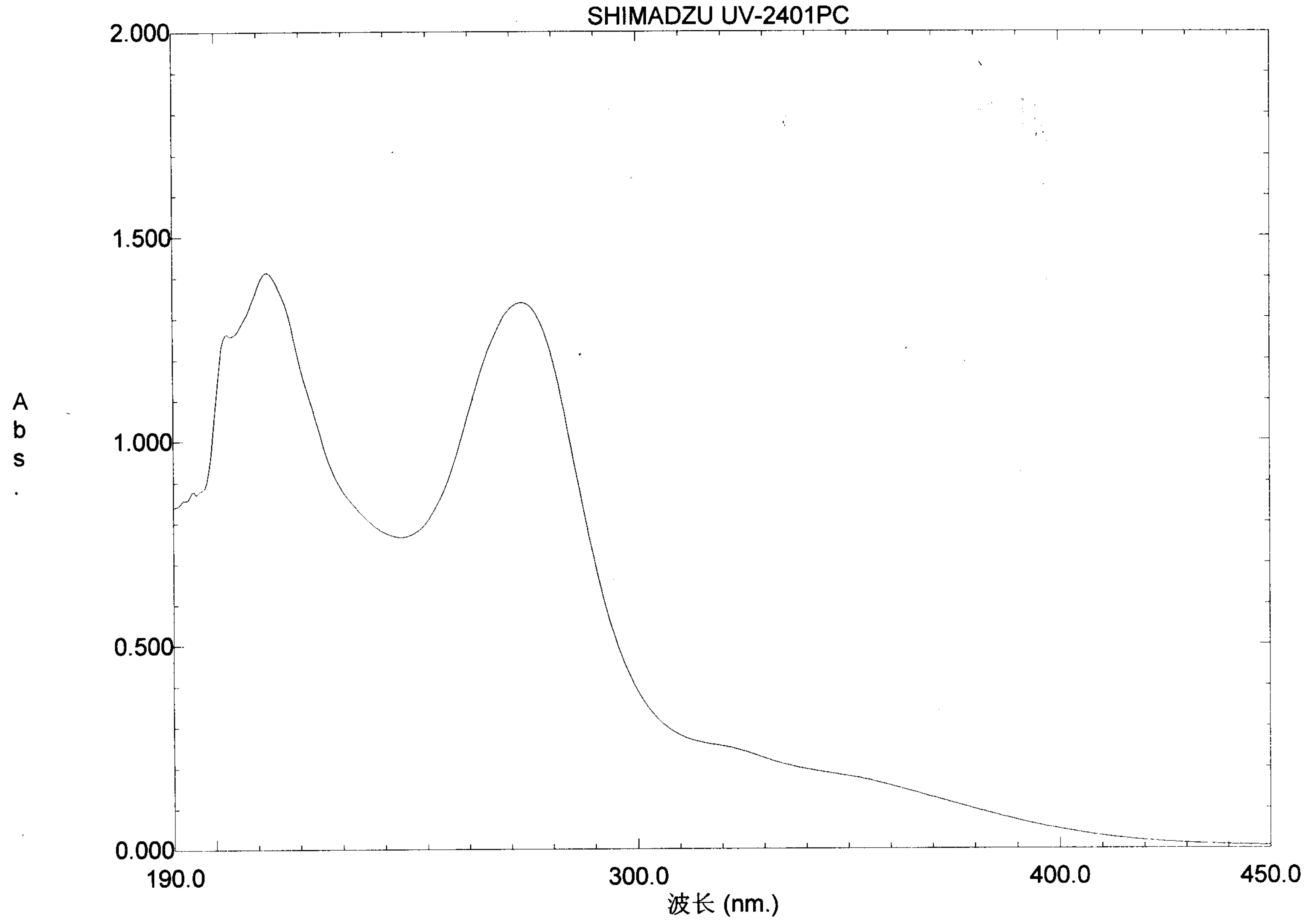

文件名: WTT-3

WTT-3 ———

样品浓度：0.010毫克/毫升
溶剂：甲醇

创建于: 14:23 08-12-25
数据: 原始

测量模式: Abs.
扫描速度: 中速
狭缝: 2.0
采样间隔: 0.5

否.	波长 (nm.)	Abs.
1	880.00	-0.0039
2	273.00	1.3388
3	212.50	1.4125

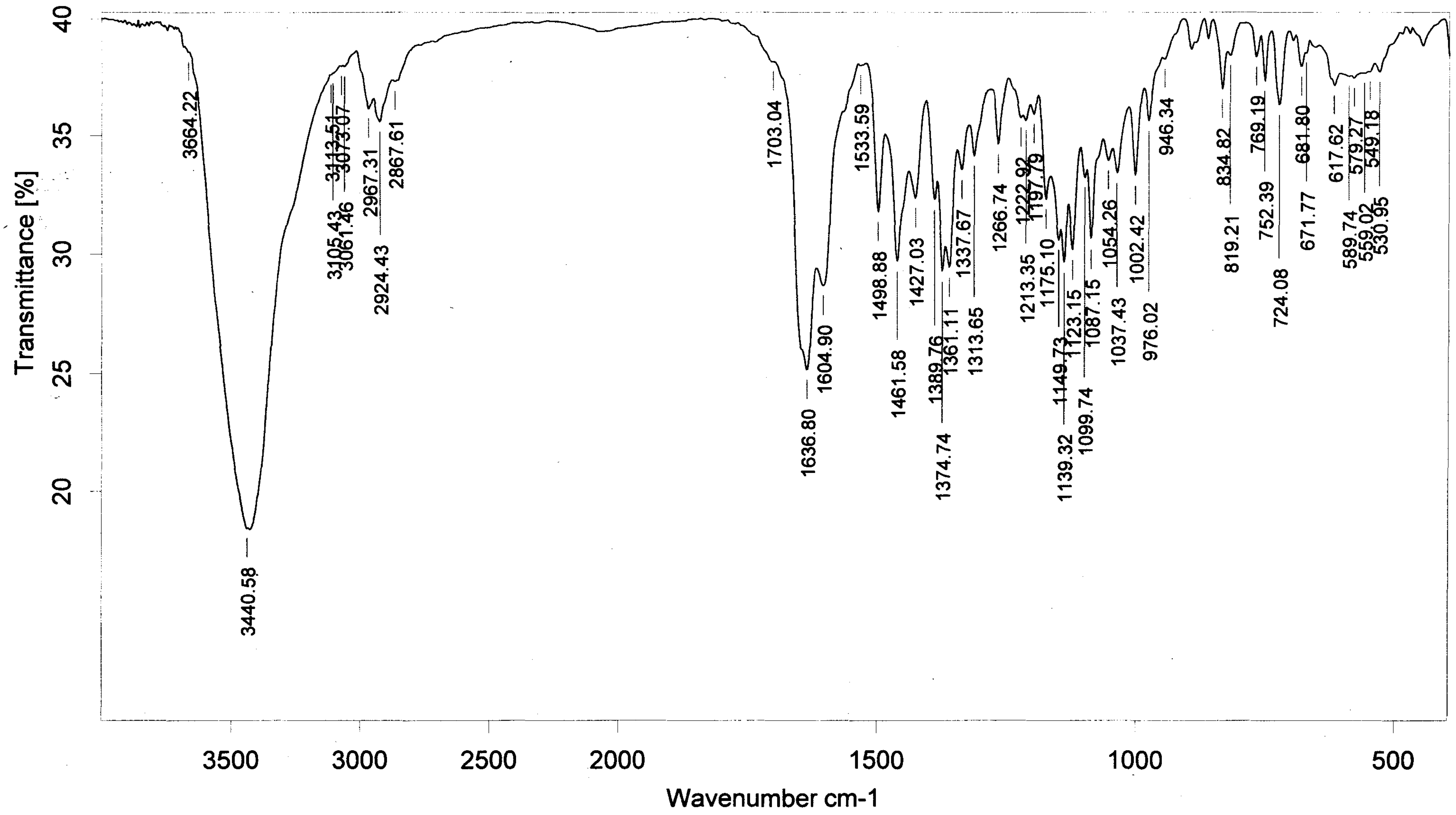

Sample : Wtt-3	Frequency Range : 399.271 - 3996.57		Measured on : 30/12/2008
Technique : KBr压片	Resolution : 4	Instrument : Tensor27	Sample Scans : 16
Customer : 081230IR1	Zerofilling : 2	Acquisition : Double Sided,For	

Bruker Drx-500MHz; wtt11 H

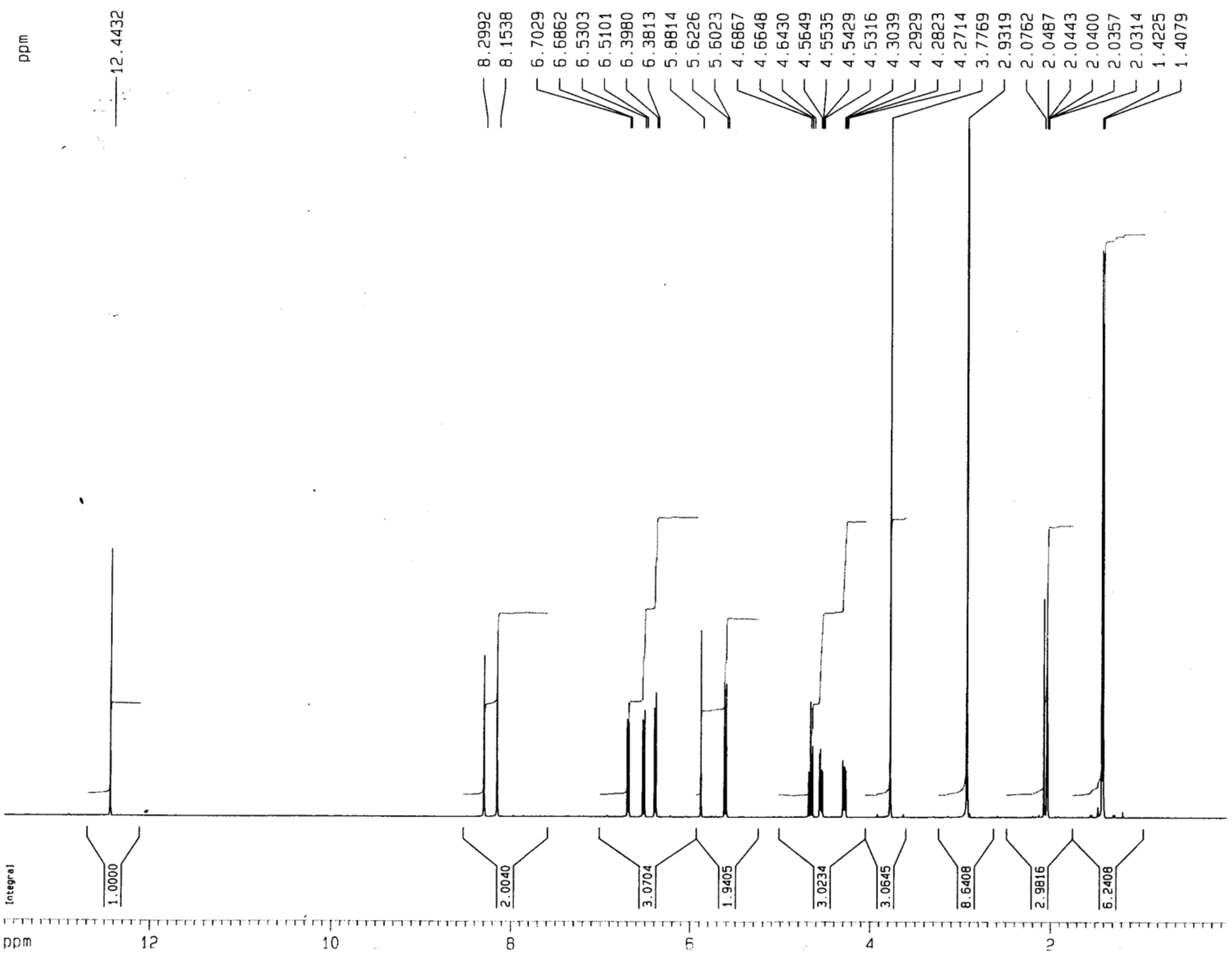

Current Data Parameters

NAME	wtt11
EXPNO	21
PROCNO	1

F2 - Acquisition Parameters

Date_	20080516
Time	13.08
INSTRUM	spect
PROBHD	5 mm DUL 13C-1
PULPROG	zg
TD	32768
SOLVENT	CDCl3
NS	1
DS	0
SWH	12019.230 Hz
FIDRES	0.366798 Hz
AQ	1.3631988 sec
RG	161.3
DW	41.600 usec
DE	6.00 usec
TE	0.0 K
D1	1.00000000 sec
MCREST	0.00000000 sec
MCWRK	0.01500000 sec

======== CHANNEL f1 ========

NUC1	1H
P1	9.20 usec
PL1	-1.00 dB
SFO1	500.0330002 MHz

F2 - Processing parameters

SI	16384
SF	500.0306428 MHz
WDW	GM
SSB	0
LB	-0.40 Hz
GB	0.2
PC	1.00

1D NMR plot parameters

CX	22.00 cm
CY	43.00 cm
F1P	13.615 ppm
F1	6807.88 Hz
F2P	0.037 ppm
F2	18.69 Hz
PPMCM	0.61716 ppm/cm
HZCM	308.59943 Hz/cm

wtt11 c13

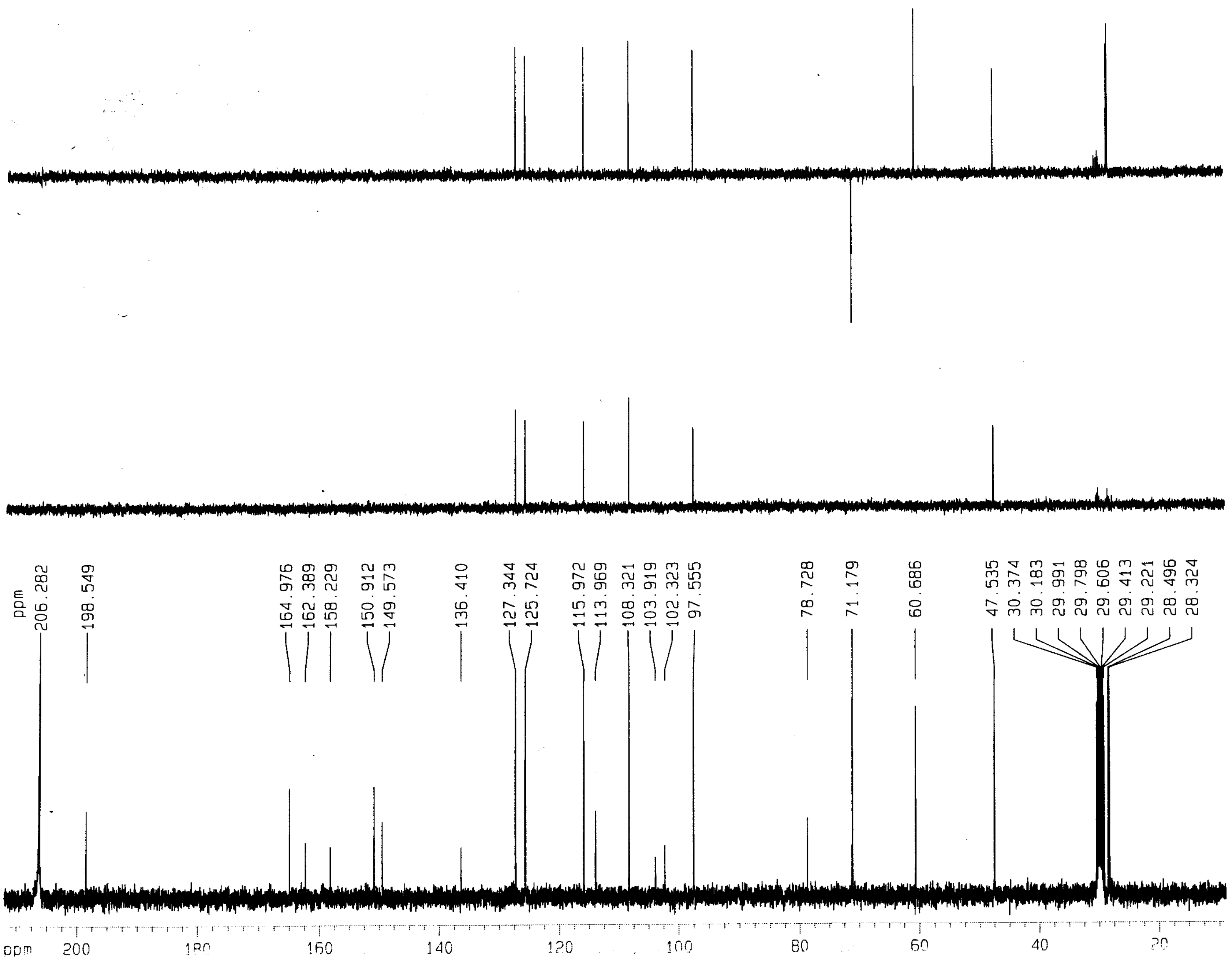

Current Data Parameters

NAME	wtt11
EXPNO	2
PROCNO	1

F2 - Acquisition Parameters

Date_	20080516
Time	13.58
INSTRUM	av400
PROBHD	5 mm QNP 1H/15
PULPROG	zgdc
TD	32768
SOLVENT	Acetone
NS	226
DS	2
SWH	23584.906 Hz
FIDRES	0.719754 Hz
AQ	0.6947316 sec
RG	32
DW	21.200 usec
DE	6.00 usec
TE	292.2 K
D1	3.00000000 sec
d11	0.03000000 sec
MCREST	0.00000000 sec
MCWRK	0.01500000 sec

======== CHANNEL f1 ========

NUC1	13C
P1	9.40 usec
PL1	-4.00 dB
SFO1	100.6239976 MHz

======== CHANNEL f2 ========

CPDPRG2	waltz16
NUC2	1H
PCPD2	90.00 usec
PL2	-3.00 dB
PL12	14.00 dB
SFO2	400.1316005 MHz

F2 - Processing parameters

SI	32768
SF	100.6126869 MHz
WDW	EM
SSB	0
LB	1.00 Hz
GB	0
PC	1.50

1D NMR plot parameters

CX	22.00 cm
CY	6.00 cm
F1P	212.000 ppm
F1	21329.89 Hz
F2P	9.000 ppm
F2	905.51 Hz
PPMCM	9.22727 ppm/cm
HZCM	928.38068 Hz/cm

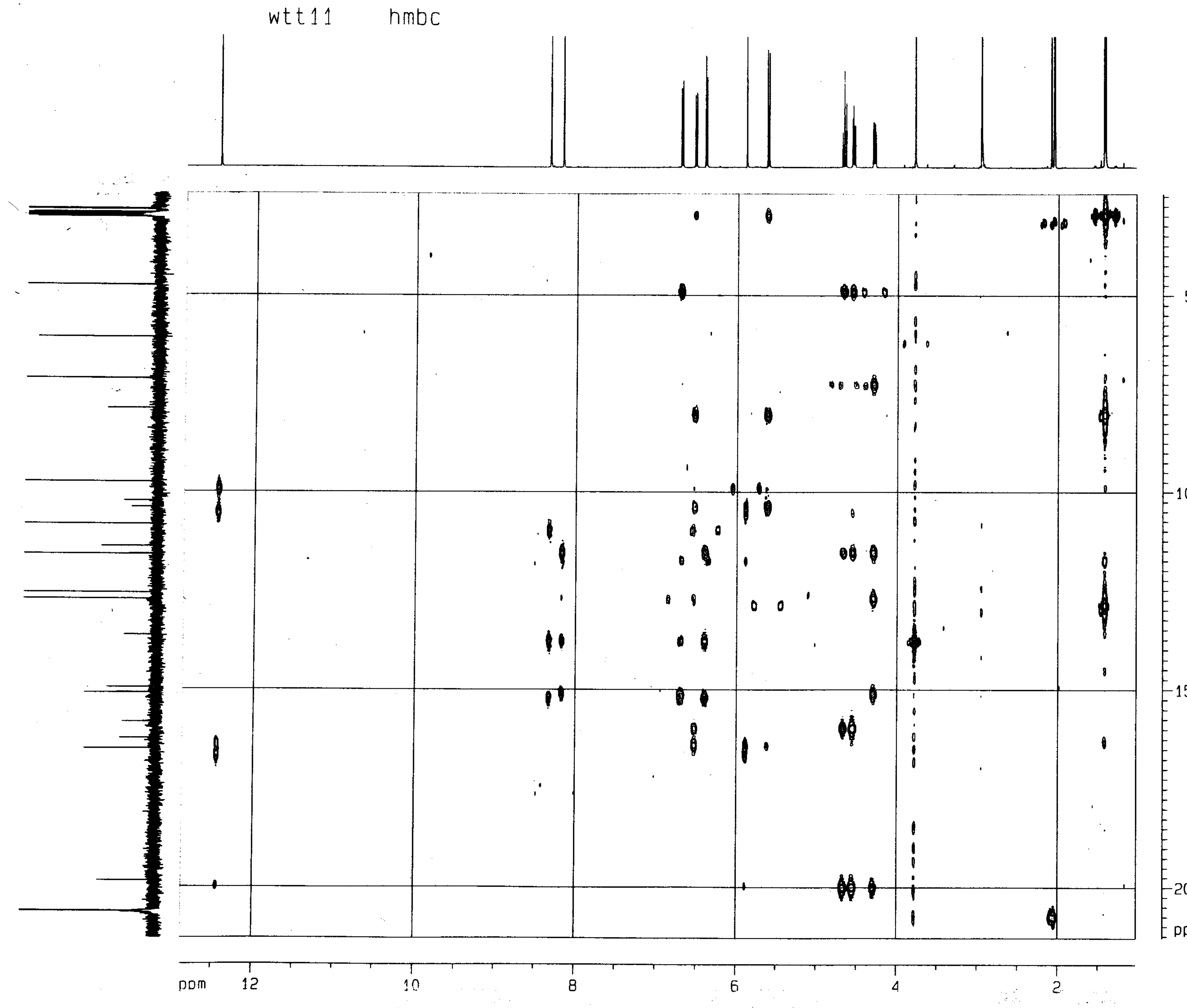

Current Data Parameters

NAME	wtt11	
EXPNO	27	
PROCNO	1	

F2 - Acquisition Parameters

Date_	20080522	
Time	10.54	
INSTRUM	spect	
PROBHD	5 mm BBI 1H-BB	
PULPROG	inv4gplplrndqf	
TD	2048	
SOLVENT	MeOD	
NS	8	
DS	16	
SWH	6510.417	Hz
FIDRES	3.178914	Hz
AQ	0.1573364	sec
RG	16384	
DW	76.800	usec
DE	6.00	usec
TE	0.0	K
CNST2	145.0000000	
d0	0.00000300	sec
D1	1.39999998	sec
d2	0.00344828	sec
D6	0.08340000	sec
d13	0.00000400	sec
D16	0.00020000	sec
IN0	0.00001988	sec
MCREST	0.00000000	sec
MCWRK	1.39999998	sec

======== CHANNEL f1 ========

NUC1	1H	
P1	9.20	usec
p2	18.40	usec
PL1	-1.00	dB
SFO1	500.0335002	MHz

======== CHANNEL f2 ========

NUC2	13C	
P3	12.00	usec
PL2	-1.00	dB
SFO2	125.7477319	MHz

====== GRADIENT CHANNEL =====

GPNAM1	SINE.100	
GPNAM2	SINE.100	
GPNAM3	SINE.100	
GPX1	0.00	%
GPX2	0.00	%
GPX3	0.00	%
GPY1	0.00	%
GPY2	0.00	%
GPY3	0.00	%
GPZ1	50.00	%
GPZ2	30.00	%
GPZ3	40.10	%
P16	1000.00	usec

F1 - Acquisition parameters

ND0	2	
TD	128	
SFO1	125.7467	MHz
FIDRES	196.540878	Hz
SW	200.063	ppm
FnMODE	QF	

F2 - Processing parameters

SI	1024	
SF	500.0300080	MHz
WDW	SINE	
SSB	0	
LB	0.00	Hz
GB	0	
PC	1.40	

F1 - Processing parameters

SI	512	
MC2	QF	
SF	125.7313431	MHz
WDW	SINE	
SSB	0	
LB	0.00	Hz
GB	0	

2D NMR plot parameters

CX2	18.00	cm
CX1	14.00	cm
F2PLO	12.871	ppm
F2LO	6435.89	Hz
F2PHI	1.033	ppm
F2HI	516.75	Hz
F1PLO	213.013	ppm
F1LO	26782.38	Hz
F1PHI	24.259	ppm
F1HI	3050.06	Hz
F2PPMCM	0.65764	ppm/cm
F2HZCM	328.84103	Hz/cm
F1PPMCM	13.48244	ppm/cm
F1HZCM	1695.16516	Hz/cm

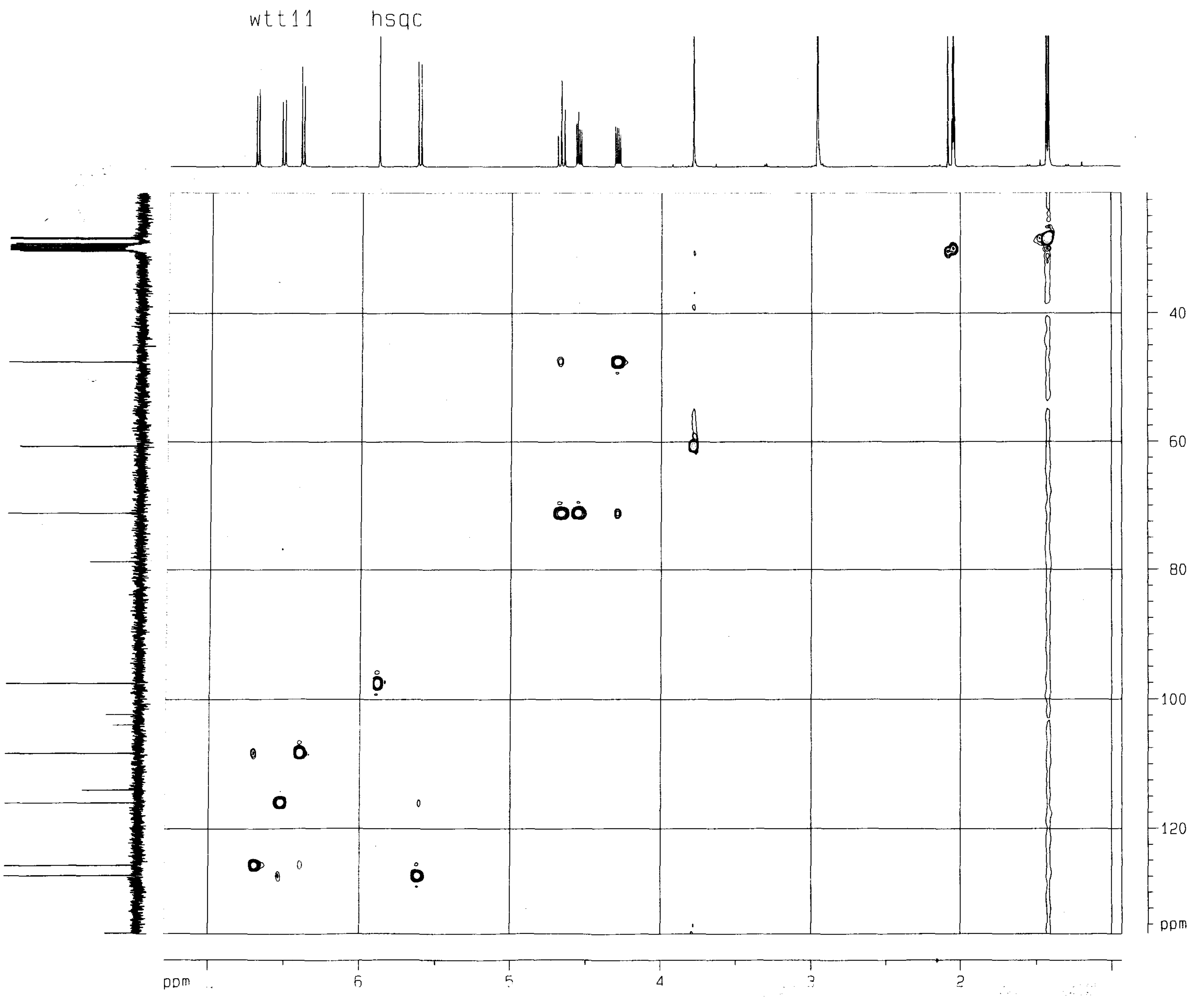

Current Data Parameters

NAME	wtt11	
EXPNO	26	
PROCNO	1	

F2 - Acquisition Parameters

Date_	20080522	
Time	10.46	
INSTRUM	spect	
PROBHD	5 mm BBI 1H-BB	
PULPROG	hsqcetgpsi2	
TD	1024	
SOLVENT	MeOD	
NS	2	
DS	16	
SWH	6510.417	Hz
FIDRES	6.357829	Hz
AQ	0.0786932	sec
RG	8192	
DW	76.800	usec
DE	6.00	usec
TE	0.0	K
CNST2	145.0000000	
d0	0.00000300	sec
D1	1.50000000	sec
d4	0.00172414	sec
d11	0.03000000	sec
d13	0.00000400	sec
D16	0.00020000	sec
D24	0.00086207	sec
DELTA	0.00127440	sec
DELTA1	0.00120800	sec
DELTA2	0.00006207	sec
DELTA3	0.00052414	sec
IN0	0.00003059	sec
MCREST	0.00000000	sec
MCWRK	0.25000051	sec
ST1CNT	64	

======== CHANNEL f1 ========

NUC1	1H	
P1	9.20	usec
p2	18.40	usec
P28	2000.00	usec
PL1	-1.00	dB
SFO1	500.0335002	MHz

======== CHANNEL f2 ========

CPDPRG2	garp	
NUC2	13C	
P3	12.00	usec
p4	24.00	usec
PCPD2	75.00	usec
PL2	-1.00	dB
PL12	15.50	dB
SFO2	125.7420739	MHz

====== GRADIENT CHANNEL =====

GPNAM1	SINE.100	
GPNAM2	SINE.100	
GPNAM3	SINE.100	
GPNAM4	SINE.100	
GPX1	0.00	%
GPX2	0.00	%
GPX3	0.00	%
GPX4	0.00	%
GPY1	0.00	%
GPY2	0.00	%
GPY3	0.00	%
GPY4	0.00	%
GPZ1	80.00	%
GPZ2	20.10	%
GPZ3	11.00	%
GPZ4	-5.00	%
P16	1000.00	usec
P19	600.00	usec

F1 - Acquisition parameters

ND0	2	
TD	128	
SFO1	125.7425	MHz
FIDRES	127.707397	Hz
SW	130.000	ppm
FnMODE	Echo-Antiecho	

F2 - Processing parameters

SI	1024	
SF	500.0300080	MHz
WDW	QSINE	
SSB	2	
LB	0.00	Hz
GB	0	
PC	1.40	

F1 - Processing parameters

SI	1024	
MC2	echo-antiecho	
SF	125.7329176	MHz
WDW	QSINE	
SSB	2	
LB	0.00	Hz
GB	0	

2D NMR plot parameters

CX2	18.00	cm
CX1	14.00	cm
F2PLO	7.289	ppm
F2LO	3644.80	Hz
F2PHI	0.932	ppm
F2HI	465.88	Hz
F1PLO	136.512	ppm
F1LO	17164.07	Hz
F1PHI	21.357	ppm
F1HI	2685.25	Hz
F2PPMCM	0.35319	ppm/cm
F2HZCM	176.6063[illegible]	[illegible]
F1PPMCM	[illegible]	[illegible]
F1HZCM	[illegible]	[illegible]

D:\LUODX\2007-06-06\wtt11
MeOH (95):Water((5)0.5% formic acid)
wtt11 #21-25 RT: 0.33-0.39 AV: 5 NL: 3.70E6
T: + c Full ms [200.00-1000.00]

2008-05-16 00:43:22

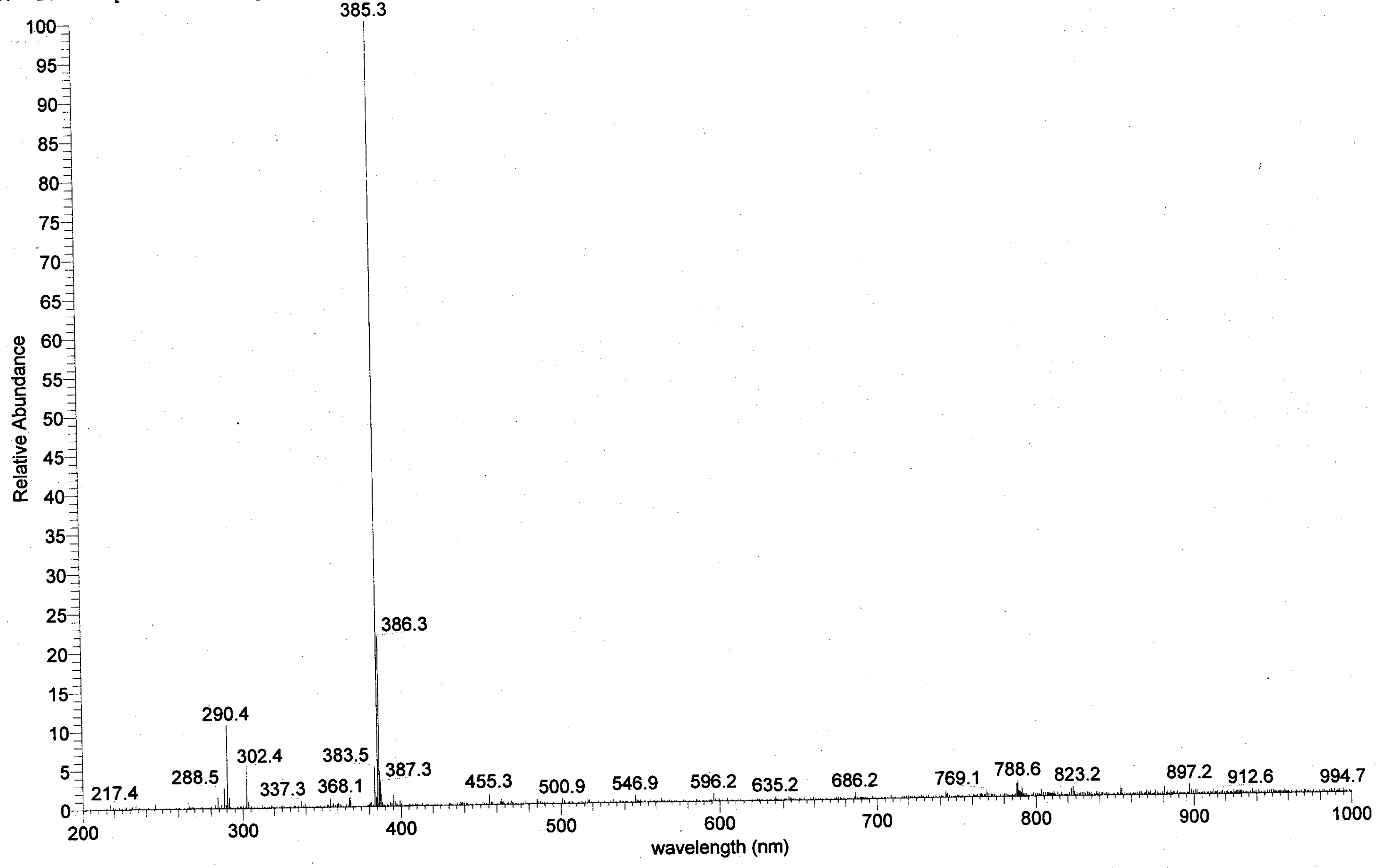

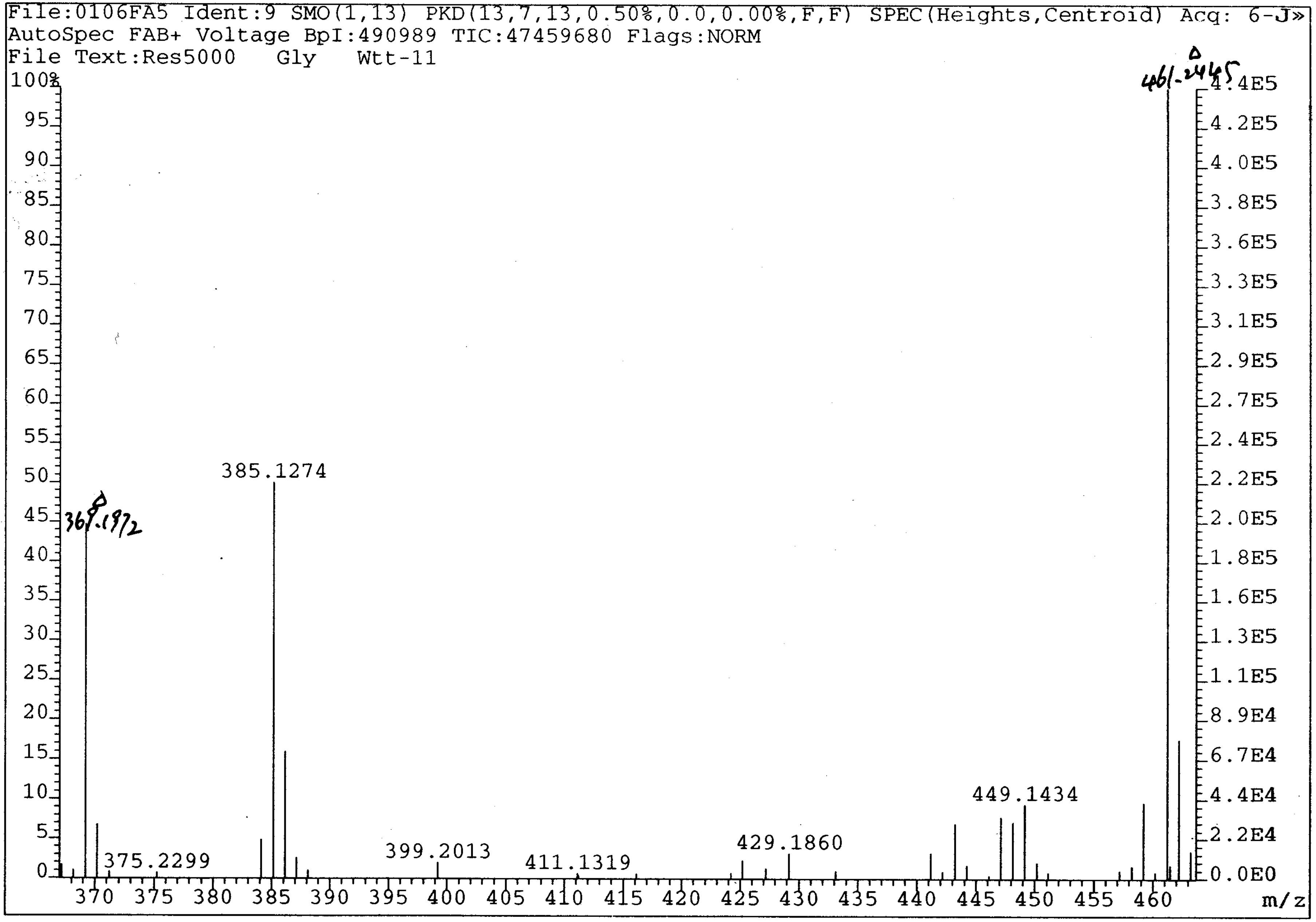
File:0106FA5 Ident:9 SMO(1,13) PKD(13,7,13,0.50%,0.0,0.00%,F,F) SPEC(Heights,Centroid) Acq: 6-J»
AutoSpec FAB+ Voltage BpI:490989 TIC:47459680 Flags:NORM
File Text:Res5000 Gly Wtt-11
461.2445
369.1972
385.1274
375.2299
399.2013
411.1319
429.1860
449.1434
m/z

File:0106FA5 Ident:9 SMO(1,13) PKD(13,7,13,0.50%,0.0,0.00%,F,F)
AutoSpec FAB+ Voltage BpI:490989 TIC:47459680 Flags:NORM
File Text:Res5000 Gly Wtt-11
Heteroatom Max: 60 Ion: Both Even and Odd
Limits:

				-0.5	0	0	0
385.127388	10.0			20.0	200	400	10

Mass	mDa	PPM	Calc. Mass	DBE	C	H	O
385.127388	1.3	3.5	385.128728	11.5	21	21	7

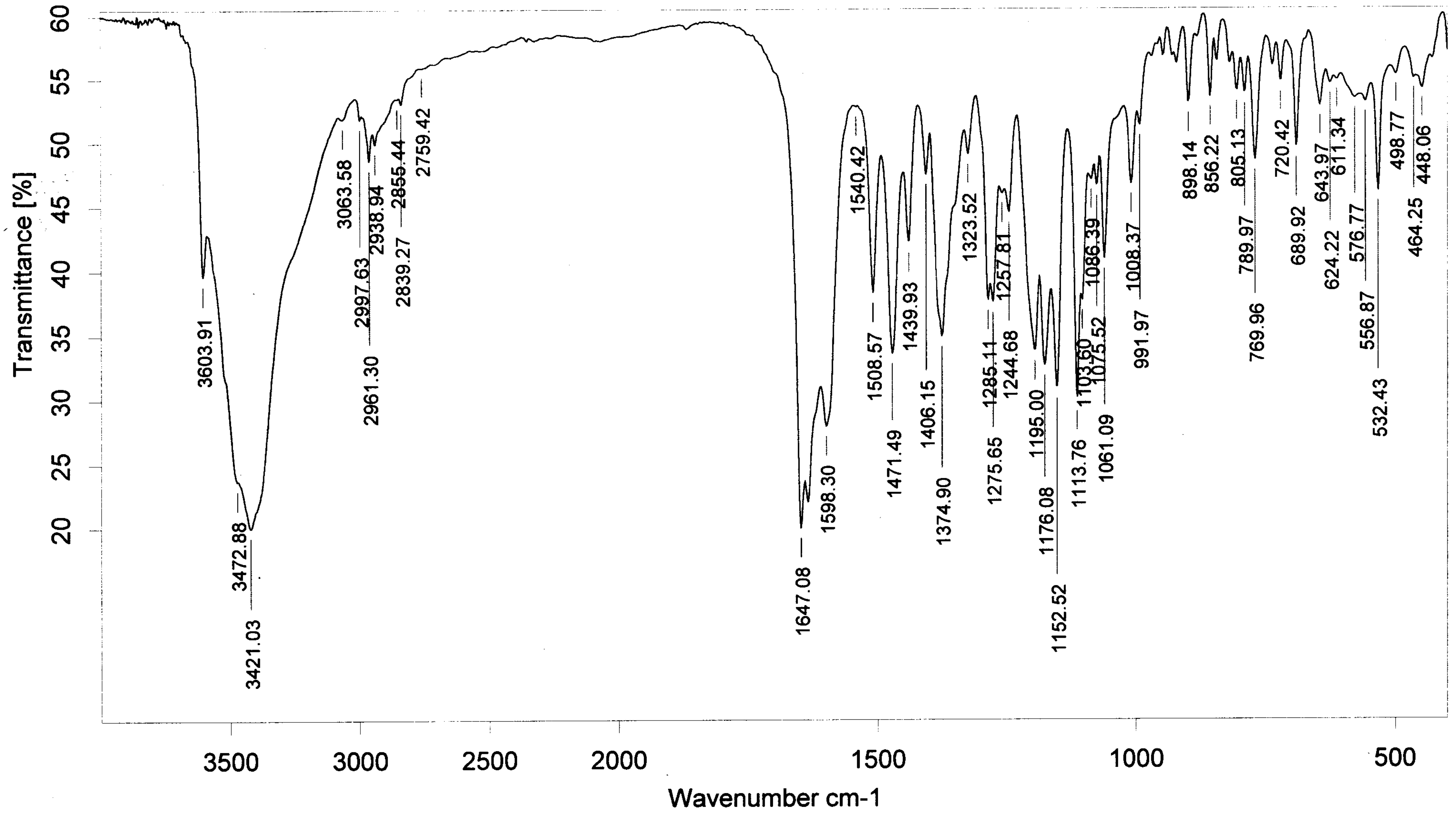

Sample : Wtt-11	Frequency Range : 399.271 - 3996.57		Measured on : 30/12/2008
Technique : KBr压片	Resolution : 4	Instrument : Tensor27	Sample Scans : 16
Customer : 081230IR2	Zerofilling : 2	Acquisition : Double Sided,For	

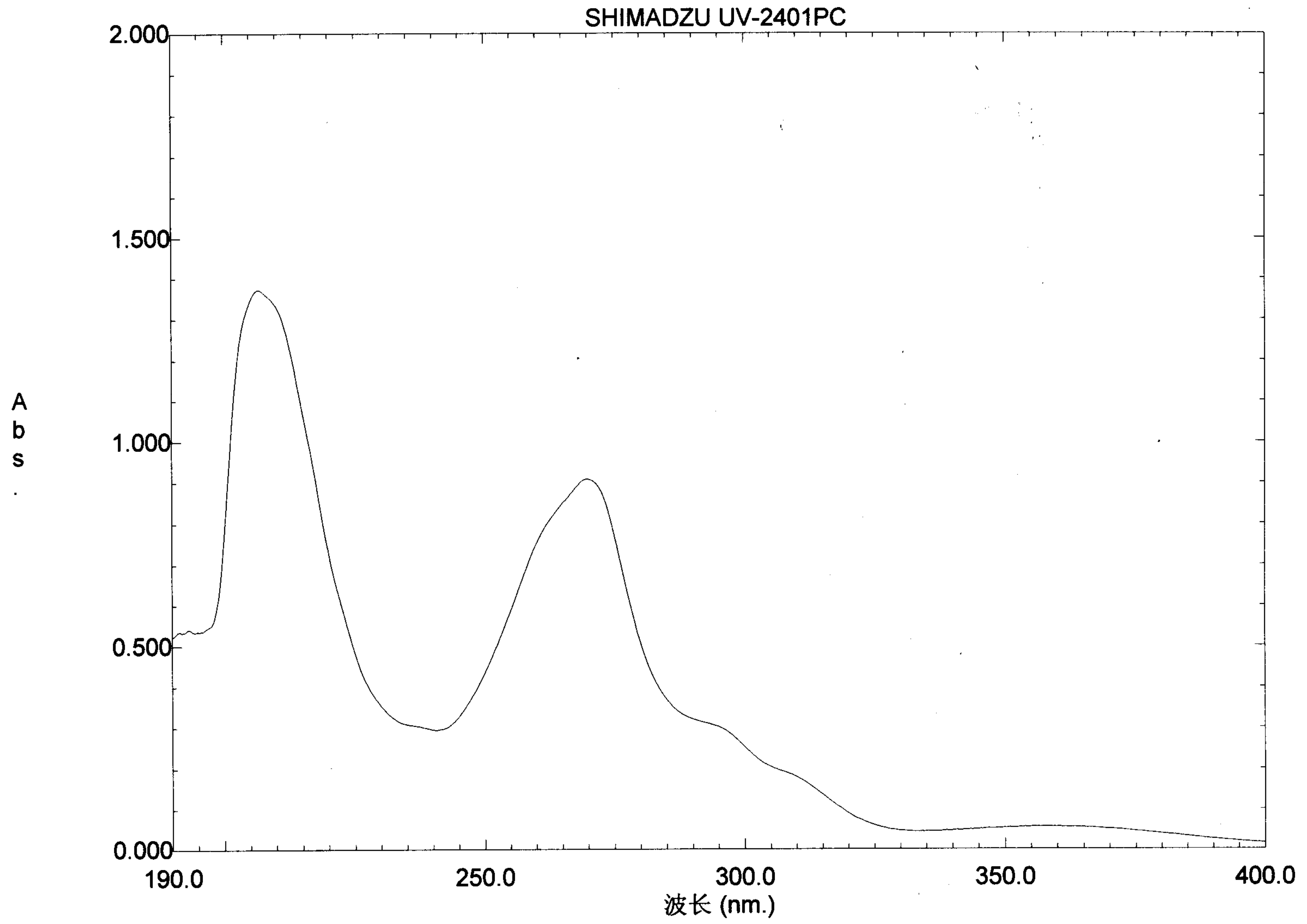

文件名: WTT-11

WTT-11 ———

样品浓度：0.010毫克/毫升
溶剂：甲醇

创建于: 14:34 08-12-25
数据: 原始

测量模式: Abs.
扫描速度: 中速
狭缝: 2.0
采样间隔: 0.5

否.	波长 (nm.)	Abs.
1	896.50	-0.0026
2	358.00	0.0553
3	270.00	0.9080
4	207.00	1.3725

wtt18 H

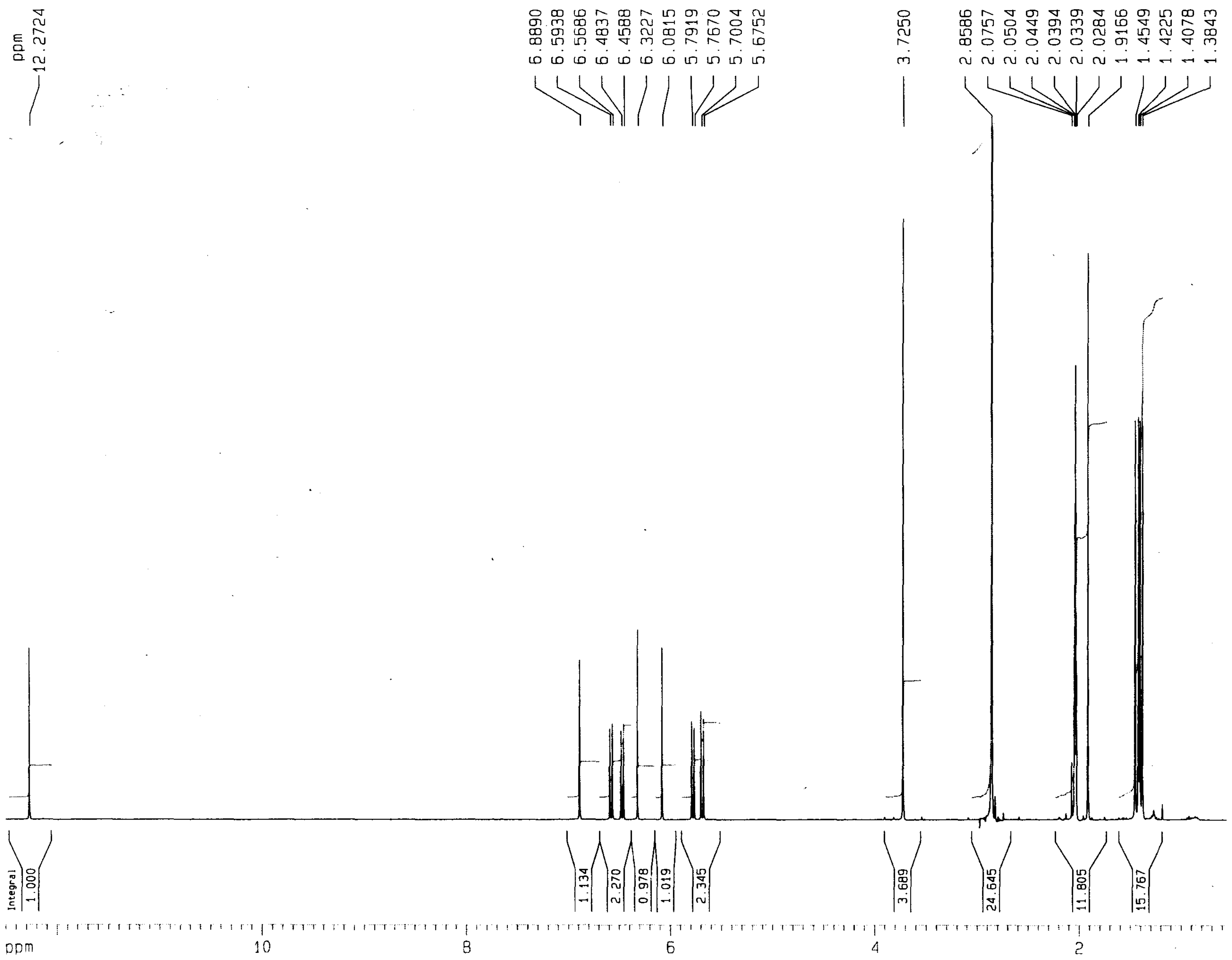

**Current Data Parameters**	
NAME	wtt18
EXPNO	1
PROCNO	1
**F2 - Acquisition Parameters**	
Date_	20080704
Time	16.45
INSTRUM	av400
PROBHD	5 mm QNP 1H/15
PULPROG	zg
TD	65536
SOLVENT	Acetone
NS	4
DS	0
SWH	6410.256 Hz
FIDRES	0.097813 Hz
AQ	5.1119361 sec
RG	114
DW	78.000 usec
DE	6.00 usec
TE	294.6 K
D1	2.00000000 sec
MCREST	0.00000000 sec
MCWRK	0.01500000 sec
**======== CHANNEL f1 ========**	
NUC1	1H
P1	10.00 usec
PL1	-3.00 dB
SFO1	400.1324008 MHz
**F2 - Processing parameters**	
SI	32768
SF	400.1305152 MHz
WDW	EM
SSB	0
LB	0.30 Hz
GB	0
PC	1.00
**1D NMR plot parameters**	
CX	22.00 cm
CY	78.00 cm
F1P	12.507 ppm
F1	5004.41 Hz
F2P	0.562 ppm
F2	224.76 Hz
PPMCM	0.54297 ppm/cm
HZCM	217.25693 Hz/cm

wtt18 c13

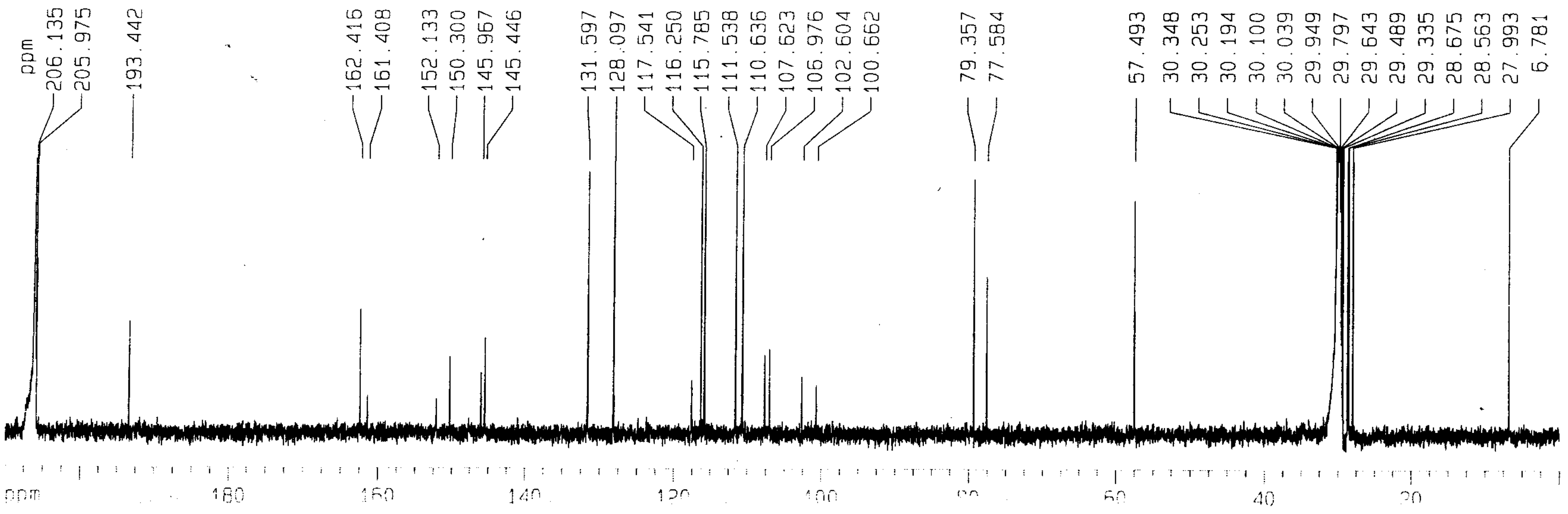

Current Data Parameters		
NAME	wtt18	
EXPNO	32	
PROCNO	1	
F2 - Acquisition Parameters		
Date_	20080708	
Time	3.45	
INSTRUM	spect	
PROBHD	5 mm DUL 13C-1	
PULPROG	zgdc	
TD	32768	
SOLVENT	Acetone	
NS	3000	
DS	0	
SWH	28985.508	Hz
FIDRES	0.884567	Hz
AQ	0.5652980	sec
RG	2048	
DW	17.250	usec
DE	6.00	usec
TE	0.0	K
D1	3.00000000	sec
d11	0.03000000	sec
MCREST	0.00000000	sec
MCWRK	0.01500000	sec
======== CHANNEL f1 ========		
NUC1	13C	
P1	5.90	usec
PL1	0.00	dB
SFO1	125.7467261	MHz
======== CHANNEL f2 ========		
CPDPRG2	waltz16	
NUC2	1H	
PCPD2	84.00	usec
PL2	-4.00	dB
PL12	18.00	dB
SFO2	500.0325001	MHz
F2 - Processing parameters		
SI	16384	
SF	125.7325343	MHz
WDW	EM	
SSB	0	
LB	1.00	Hz
GB	0	
PC	2.00	
1D NMR plot parameters		
CX	22.00	cm
CY	200.00	cm
F1P	210.000	ppm
F1	26403.83	Hz
F2P	0.000	ppm
F2	0.00	Hz
PPMCM	9.54545	ppm/cm
HZCM	1200.17407	Hz/cm

wtt18 hmbc

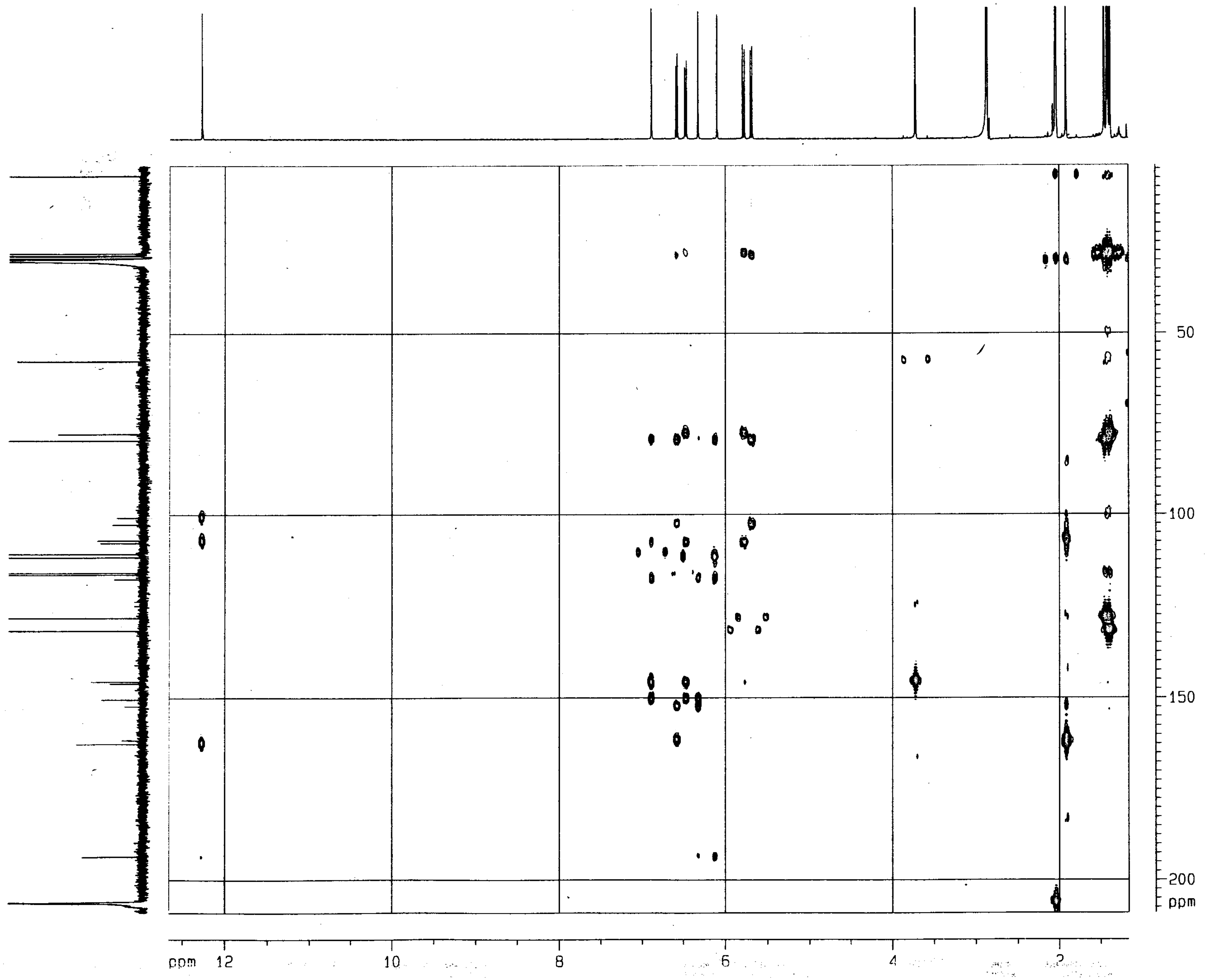

Current Data Parameters

NAME	wtt18
EXPNO	27
PROCNO	1

F2 - Acquisition Parameters

Date_	20080717
Time	19.28
INSTRUM	spect
PROBHD	5 mm BBI 1H-BB
PULPROG	inv4gplplrndqf
TD	2048
SOLVENT	Acetone
NS	64
DS	16
SWH	7507.507 Hz
FIDRES	3.665775 Hz
AQ	0.1364468 sec
RG	32768
DW	66.600 usec
DE	6.00 usec
TE	0.0 K
CNST2	145.0000000
d0	0.00000300 sec
D1	1.39999998 sec
d2	0.00344828 sec
D6	0.08340000 sec
d13	0.00000400 sec
D16	0.00020000 sec
IN0	0.00001791 sec
MCREST	0.00000000 sec
MCWRK	1.39999998 sec

======== CHANNEL f1 ========

NUC1	1H
P1	9.20 usec
p2	18.40 usec
PL1	-1.00 dB
SFO1	500.0333002 MHz

======== CHANNEL f2 ========

NUC2	13C
P3	12.00 usec
PL2	-1.00 dB
SFO2	125.7467261 MHz

====== GRADIENT CHANNEL =====

GPNAM1	SINE.100
GPNAM2	SINE.100
GPNAM3	SINE.100
GPX1	0.00 %
GPX2	0.00 %
GPX3	0.00 %
GPY1	0.00 %
GPY2	0.00 %
GPY3	0.00 %
GPZ1	50.00 %
GPZ2	30.00 %
GPZ3	40.10 %
P16	1000.00 usec

F1 - Acquisition parameters

ND0	2
TD	128
SFO1	125.7467 MHz
FIDRES	218.073975 Hz
SW	221.982 ppm
FnMODE	QF

F2 - Processing parameters

SI	1024
SF	500.0300109 MHz
WDW	SINE
SSB	0
LB	0.00 Hz
GB	0
PC	1.40

F1 - Processing parameters

SI	512
MC2	QF
SF	125.7325389 MHz
WDW	SINE
SSB	0
LB	0.00 Hz
GB	0

2D NMR plot parameters

CX2	18.00 cm
CX1	14.00 cm
F2PLO	12.663 ppm
F2LO	6331.92 Hz
F2PHI	1.168 ppm
F2HI	583.98 Hz
F1PLO	209.096 ppm
F1LO	26290.23 Hz
F1PHI	4.000 ppm
F1HI	502.98 Hz
F2PPMCM	0.63862 ppm/cm
[illegible]	[illegible] Hz/cm
F1PPMCM	[illegible] ppm/cm
F1HZCM	1841.94617 Hz/cm

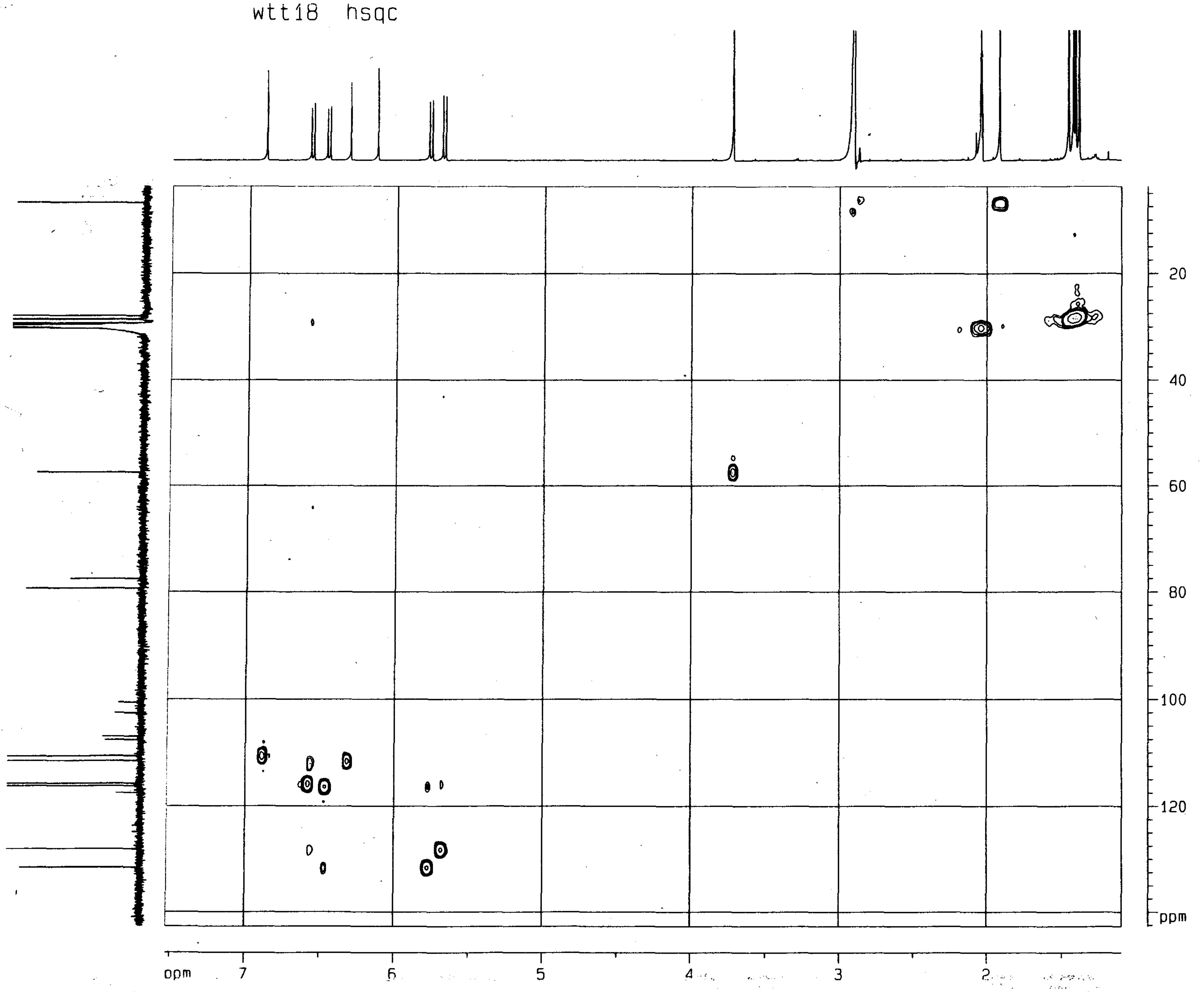

Current Data Parameters	
NAME	wtt18
EXPNO	26
PROCNO	1

F2 - Acquisition Parameters	
Date_	20080723
Time	14.37
INSTRUM	spect
PROBHD	5 mm BBI 1H-BB
PULPROG	hsqcetgpsi2
TD	1024
SOLVENT	DMSO
NS	8
DS	16
SWH	6510.417 Hz
FIDRES	6.357829 Hz
AQ	0.0786932 sec
RG	8192
DW	76.800 usec
DE	6.00 usec
TE	0.0 K
CNST2	145.0000000
d0	0.00000300 sec
D1	1.50000000 sec
d4	0.00172414 sec
d11	0.03000000 sec
d13	0.00000400 sec
D16	0.00020000 sec
D24	0.00086207 sec
DELTA	0.00127440 sec
DELTA1	0.00120800 sec
DELTA2	0.00086207 sec
DELTA3	0.00052414 sec
IN0	0.00001894 sec
MCREST	0.00000000 sec
MCWRK	0.25000051 sec
ST1CNT	128

======== CHANNEL f1 ========	
NUC1	1H
P1	9.20 usec
p2	18.40 usec
P28	2000.00 usec
PL1	-1.00 dB
SFO1	500.0333002 MHz

======== CHANNEL f2 ========	
CPDPRG2	garp
NUC2	13C
P3	12.00 usec
p4	24.00 usec
PCPD2	75.00 usec
PL2	-1.00 dB
PL12	15.50 dB
SFO2	125.7457202 MHz

====== GRADIENT CHANNEL =====	
GPNAM1	SINE.100
GPNAM2	SINE.100
GPNAM3	SINE.100
GPNAM4	SINE.100
GPX1	0.00 %
GPX2	0.00 %
GPX3	0.00 %
GPX4	0.00 %
GPY1	0.00 %
GPY2	0.00 %
GPY3	0.00 %
GPY4	0.00 %
GPZ1	80.00 %
GPZ2	20.10 %
GPZ3	11.00 %
GPZ4	-5.00 %
P16	1000.00 usec
P19	600.00 usec

F1 - Acquisition parameters	
ND0	2
TD	128
SFO1	125.7425 MHz
FIDRES	206.270630 Hz
SW	209.974 ppm
FnMODE	Echo-Antiecho

F2 - Processing parameters	
SI	1024
SF	500.0300130 MHz
WDW	QSINE
SSB	2
LB	0.00 Hz
GB	0
PC	1.40

F1 - Processing parameters	
SI	1024
MC2	echo-antiecho
SF	125.7282515 MHz
WDW	QSINE
SSB	2
LB	0.00 Hz
GB	0

2D NMR plot parameters	
CX2	18.00 cm
CX1	14.00 cm
F2PLO	7.520 ppm
F2LO	3764.04 Hz
F2PHI	1.094 ppm
F2HI	546.98 Hz
F1PLO	142.718 ppm
F1LO	17943.86 Hz
F1PHI	3.678 ppm
F1HI	462.42 Hz
F2PPMCM	0.35743 ppm/cm
F2HZCM	[illegible] Hz/cm
F1PPMCM	9.93145 ppm/cm
F1HZCM	1246.57383 Hz/cm

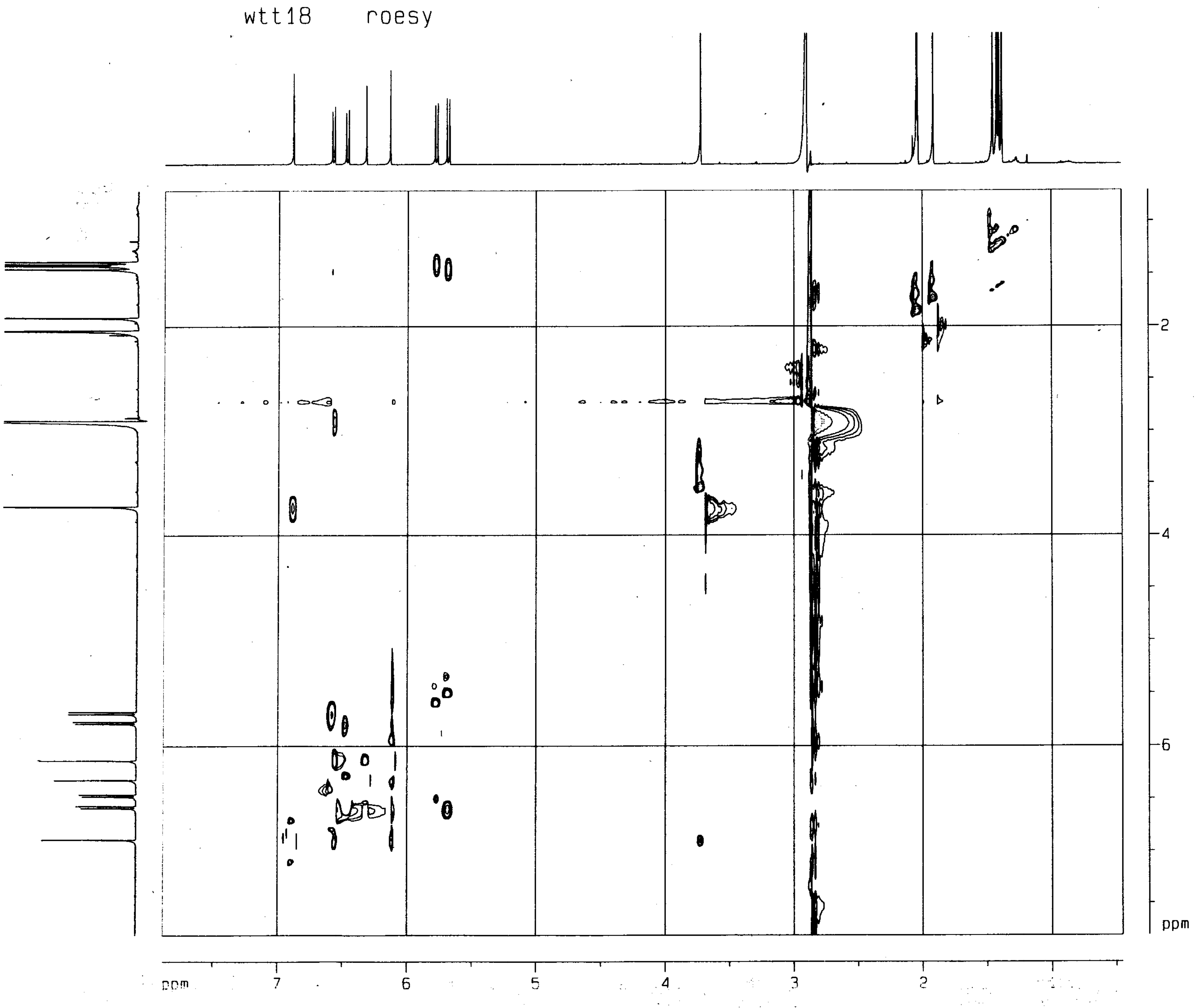

Current Data Parameters

NAME	wtt18
EXPNO	28
PROCNO	1

F2 - Acquisition Parameters

Date_	20080723
Time	13.08
INSTRUM	spect
PROBHD	5 mm BBI 1H-BB
PULPROG	croesyph
TD	1024
SOLVENT	MeOD
NS	8
DS	4
SWH	6510.417 Hz
FIDRES	6.357829 Hz
AQ	0.0786932 sec
RG	32
DW	76.800 usec
DE	6.00 usec
TE	0.0 K
d0	0.00006520 sec
D1	2.00000000 sec
d12	0.00002000 sec
d13	0.00000400 sec
IN0	0.00015384 sec
MCREST	0.00000000 sec
MCWRK	1.00000000 sec
ST1CNT	70

======== CHANNEL f1 ========

NUC1	1H
P1	9.20 usec
P15	1200000.00 usec
PL1	-1.00 dB
PL11	22.00 dB
SFO1	500.0333002 MHz

F1 - Acquisition parameters

ND0	1
TD	159
SFO1	500.0333 MHz
FIDRES	40.882801 Hz
SW	13.000 ppm
FnMODE	States-TPPI

F2 - Processing parameters

SI	1024
SF	500.0300130 MHz
WDW	QSINE
SSB	2
LB	0.00 Hz
GB	0
PC	1.00

F1 - Processing parameters

SI	1024
MC2	States-TPPI
SF	500.0300080 MHz
WDW	QSINE
SSB	2
LB	0.00 Hz
GB	0

2D NMR plot parameters

CX2	18.00 cm
CX1	14.00 cm
F2PLO	7.887 ppm
F2LO	3943.66 Hz
F2PHI	0.454 ppm
F2HI	226.77 Hz
F1PLO	7.809 ppm
F1LO	3904.74 Hz
F1PHI	0.708 ppm
F1HI	353.78 Hz
F2PPMCM	0.41296 ppm/cm
F2HZCM	206.49358 Hz/cm
F1PPMCM	0.50725 ppm/cm
F1HZCM	253.63982 Hz/cm

D:\LUODX\2007-06-06\wtt-18 2008-07-04 09:49:46
MeOH (95):Water((5)0.5% formic acid)
wtt-18 #74-75 RT: 1.23-1.25 AV: 2 NL: 7.03E6
T: + c Full ms [150.00-1000.00]

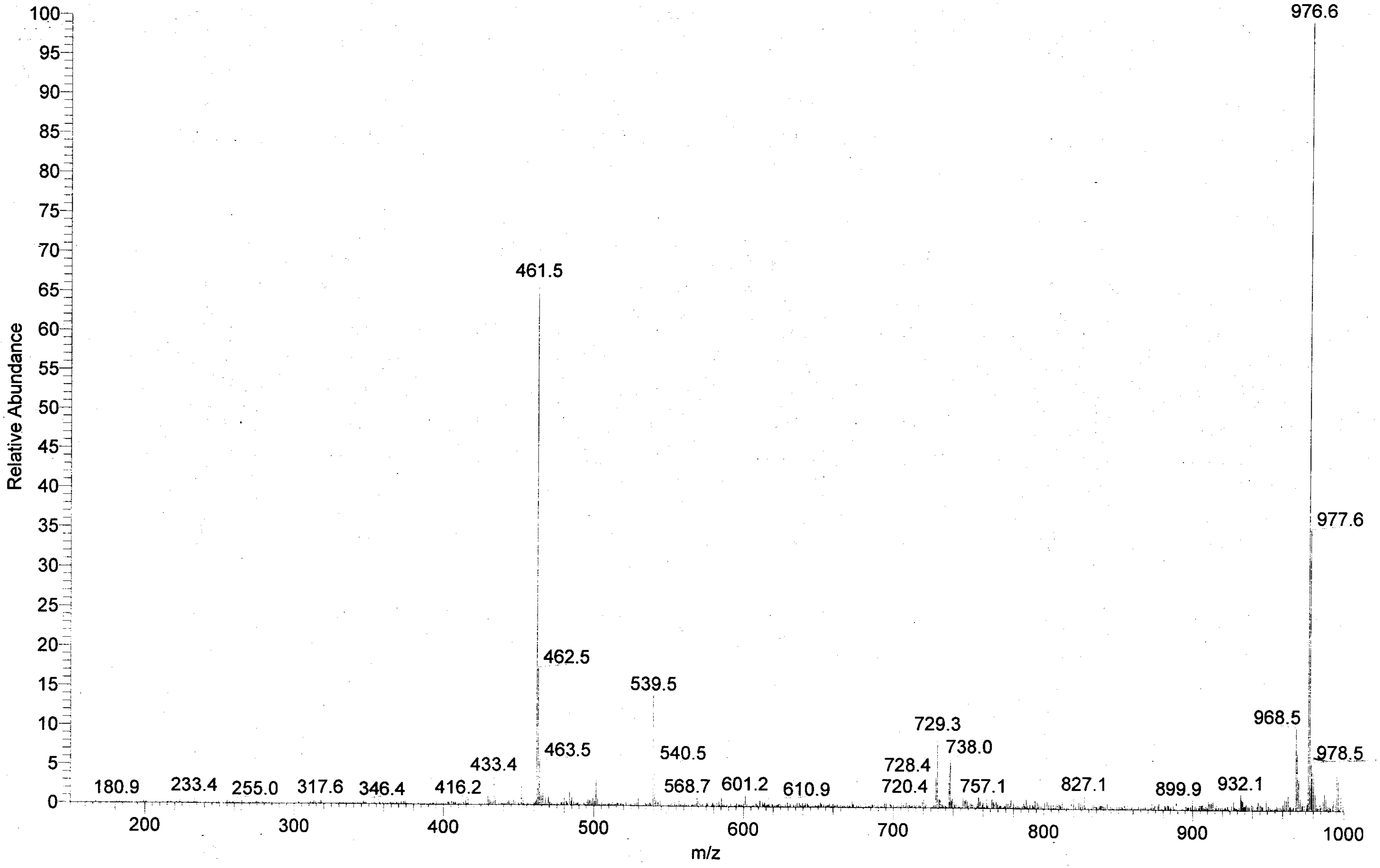

D:\LUODX\2007-06-06\wtt-18a 2008-07-10 23:20:57
MeOH (95):Water((5)0.5% formic acid)

wtt-18a #33-36 RT: 0.49-0.54 AV: 4 NL: 7.80E5
T: - c Full ms [200.00-1000.00]

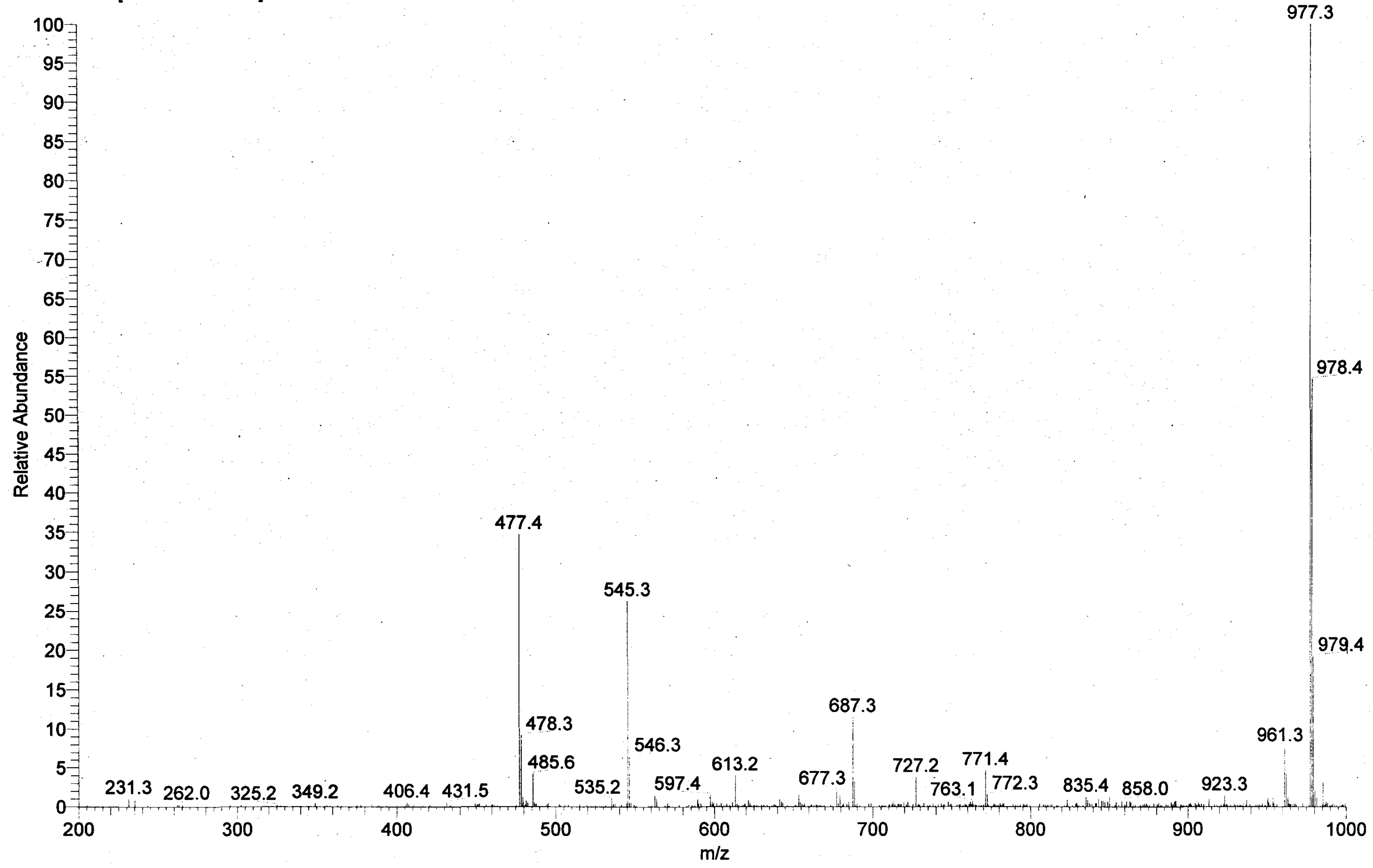

C:\Xcalibur\data\080718e-07 07/18/2008 02:50:47 PM wtt-18

080718e-07 #33-40 RT: 1.53-1.75 AV: 8 SB: 2 1.88, 1.88 NL: 6.62E6
T: + c Full ms [50.00-1000.00]

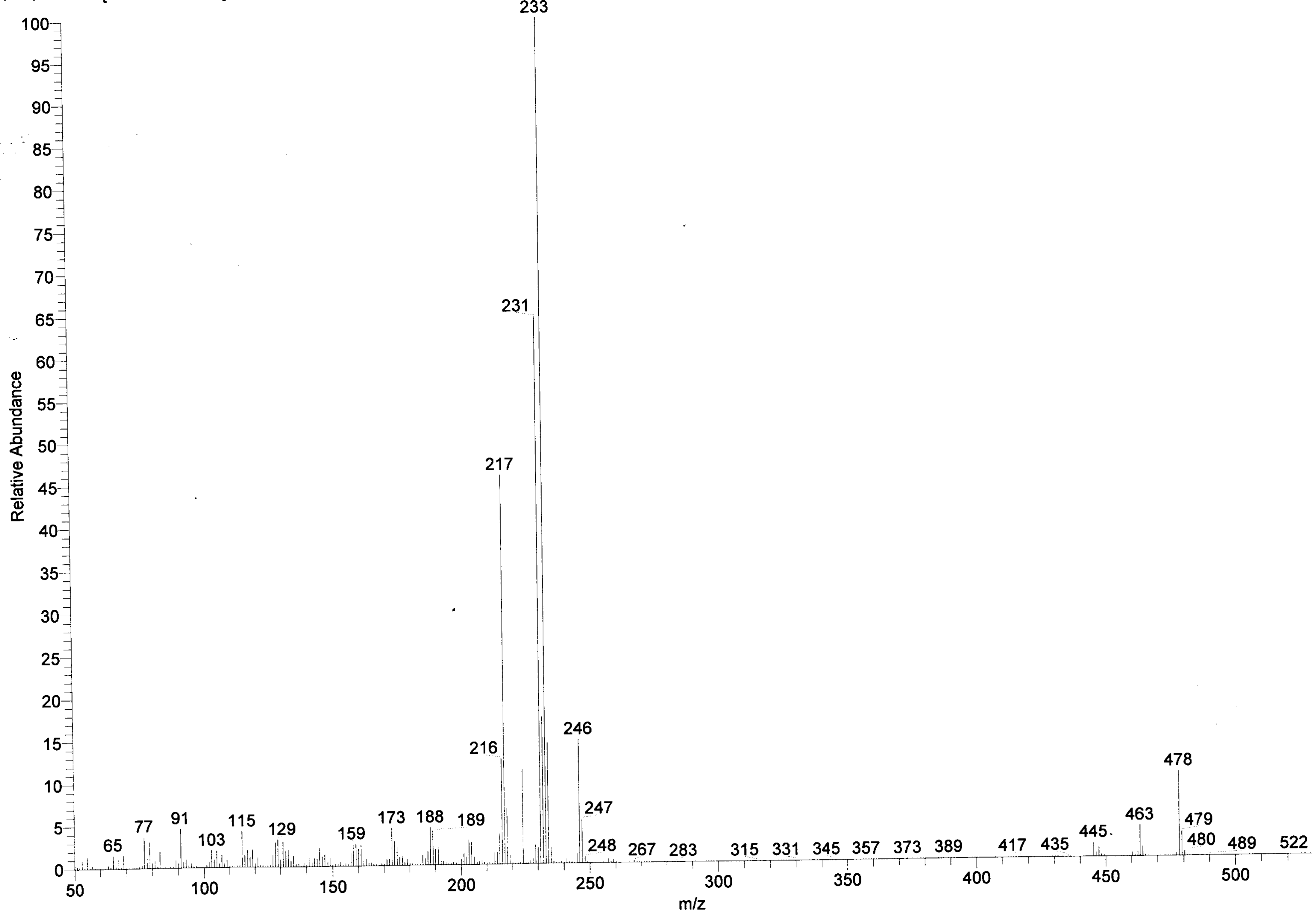

File:0106FA2 Ident:28 SMO(1,3) PKD(3,3,3,0.50%,0.0,0.00%,F,F) SPEC(Heights,Centroid) Acq: 6-JAN»
AutoSpec FAB+ Voltage BpI:333381 TIC:32700934 Flags:NORM
File Text:Res5000 Gly Wtt-18

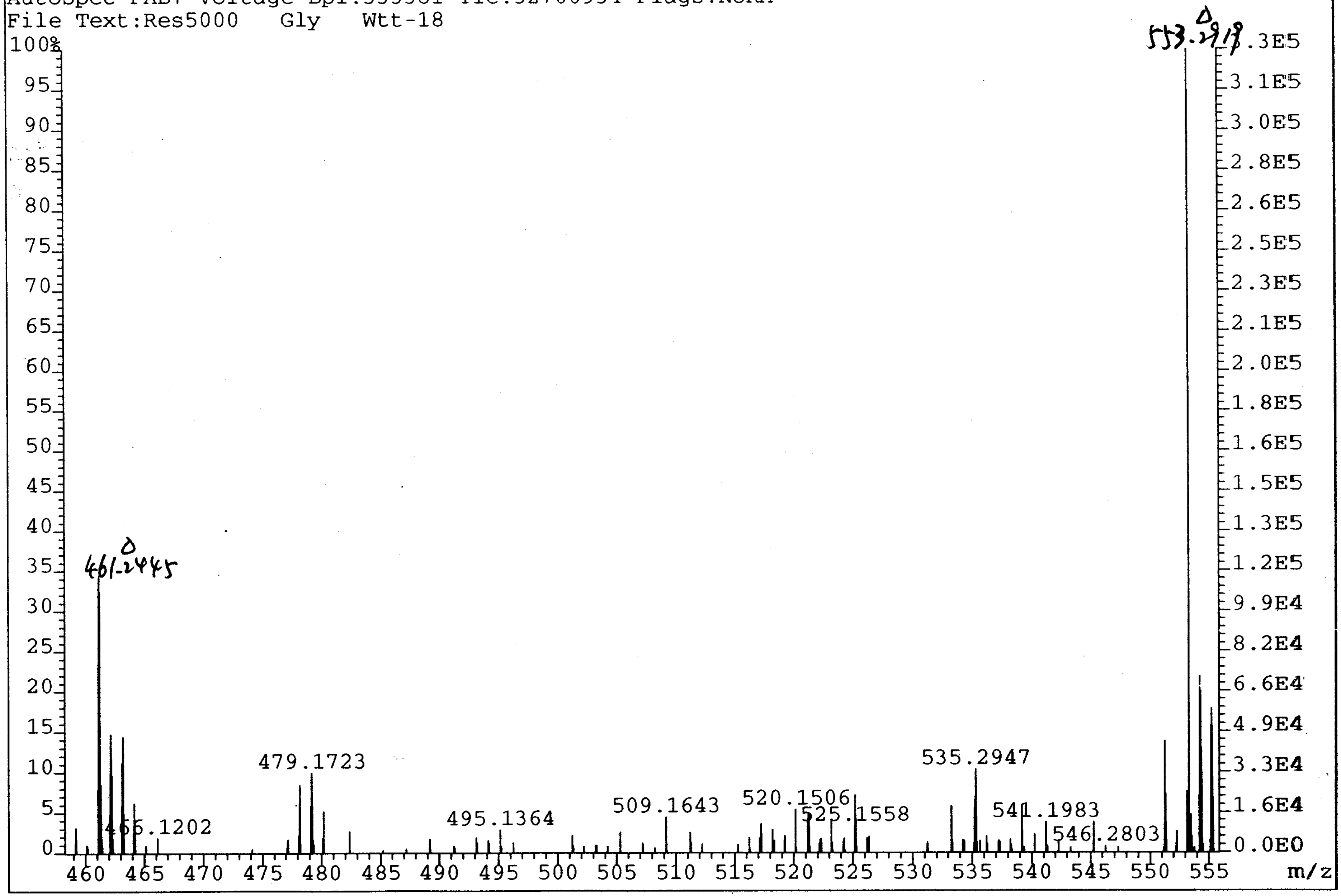

Elemental Composition Date : 6-JAN-2009

File:0106FA2 Ident:28 SMO(1,3) PKD(3,3,3,0.50%,0.0,0.00%,F,F)
AutoSpec FAB+ Voltage BpI:333381 TIC:32700934 Flags:NORM
File Text:Res5000 Gly Wtt-18
Heteroatom Max: 60 Ion: Both Even and Odd
Limits:

				-0.5	0	0	0
479.172331	10.0			20.0	200	400	10
**Mass**	**mDa**	**PPM**	**Calc. Mass**	**DBE**	**C**	**H**	**O**
479.172331	-1.7	-3.6	479.170593	14.5	27	27	8

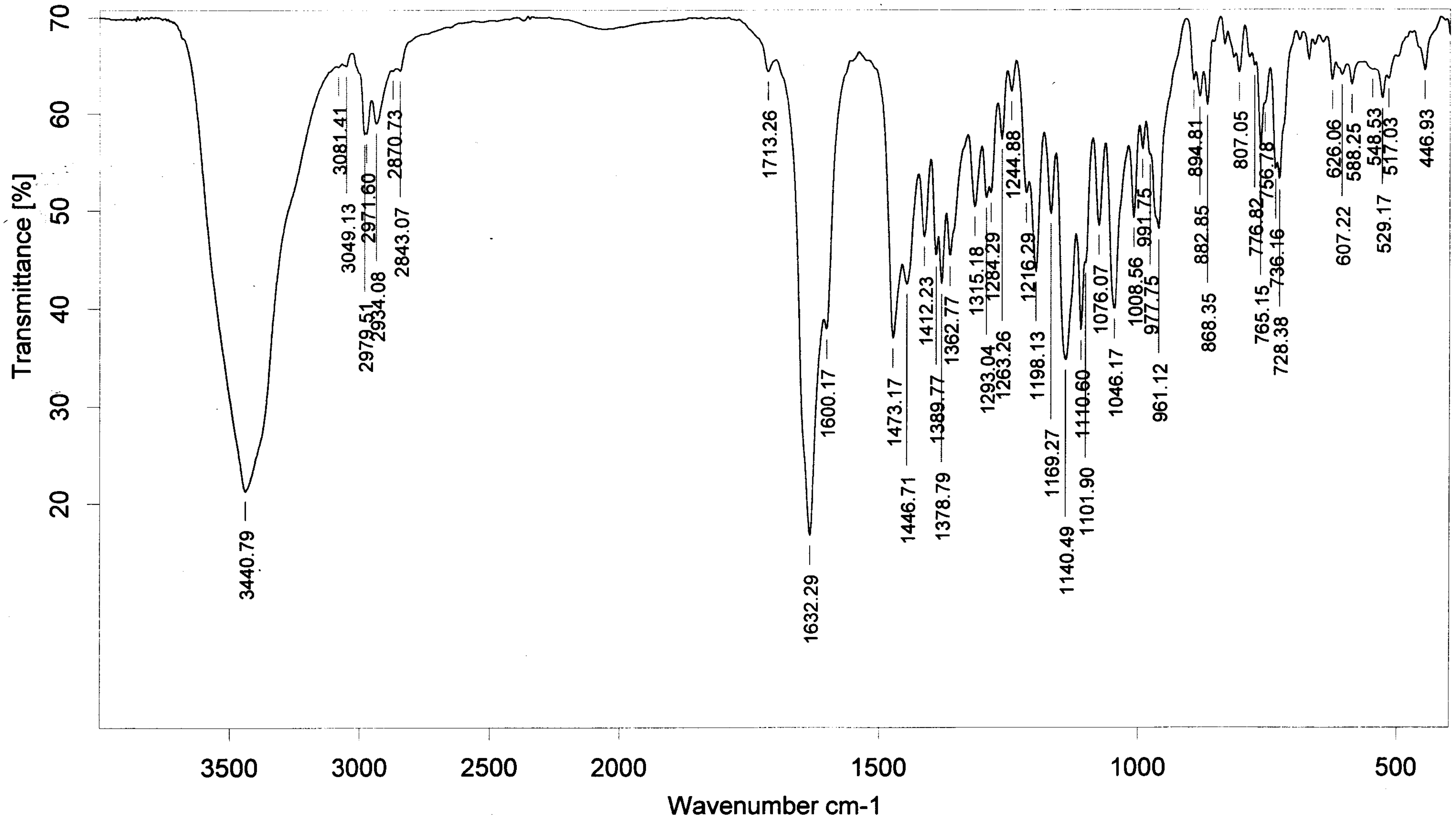

Sample : Wtt-18		Frequency Range : 399.271 - 3996.57	Measured on : 30/12/2008
Technique : KBr压片	Resolution : 4	Instrument : Tensor27	Sample Scans : 16
Customer : 081230IR4	Zerofilling : 2	Acquisition : Double Sided,For	

SHIMADZU UV-2401PC

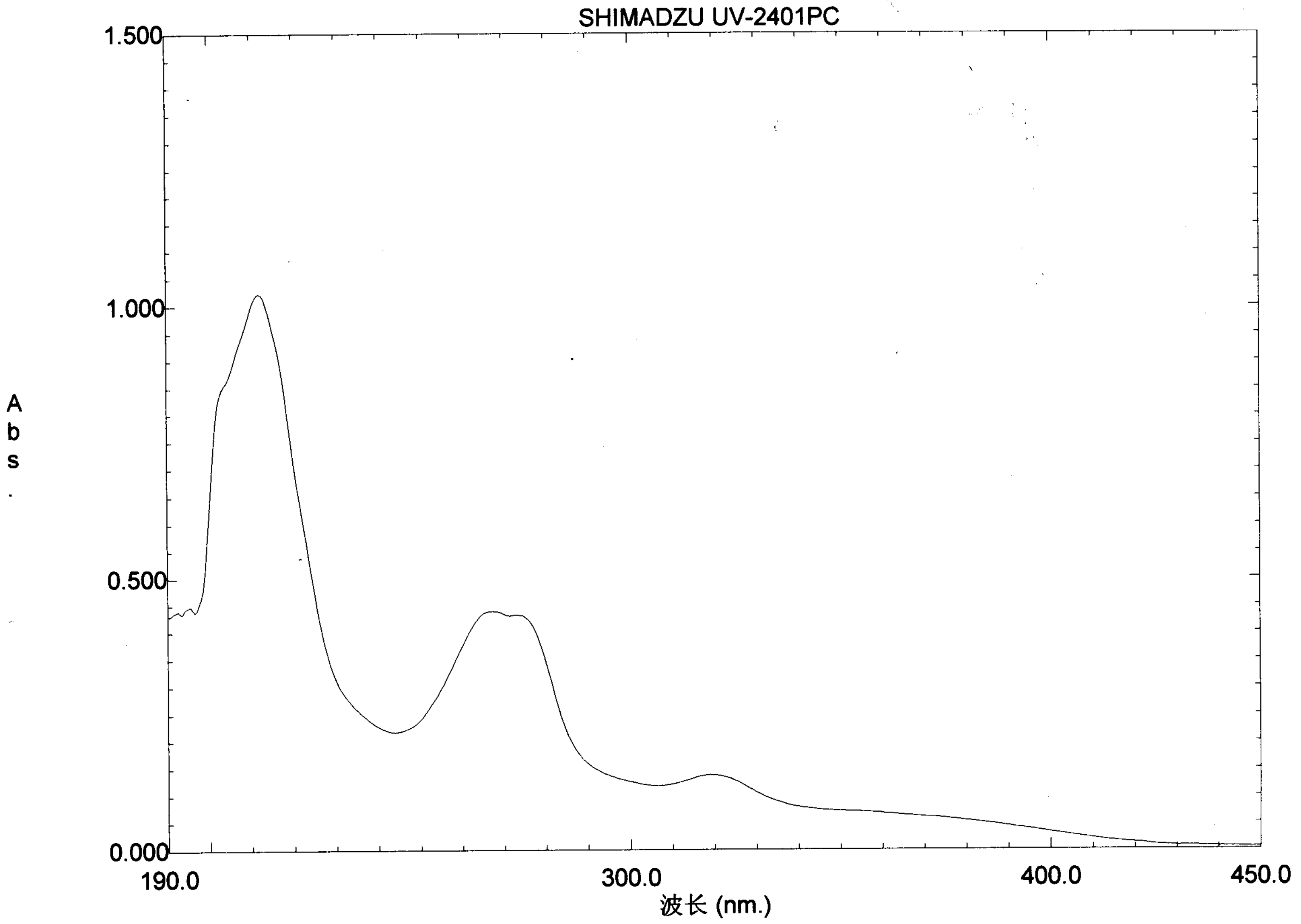

文件名: WTT-18

WTT-18 ———

样品浓度：0.010毫克/毫升
溶剂：甲醇

创建于: 14:43 08-12-25
数据: 原始

测量模式: Abs.
扫描速度: 中速
狭缝: 2.0
采样间隔: 0.5

否.	波长 (nm.)	Abs.
1	724.50	0.0009
2	319.00	0.1377
3	273.50	0.4311
4	268.00	0.4377
5	212.00	1.0224

wtt13 H

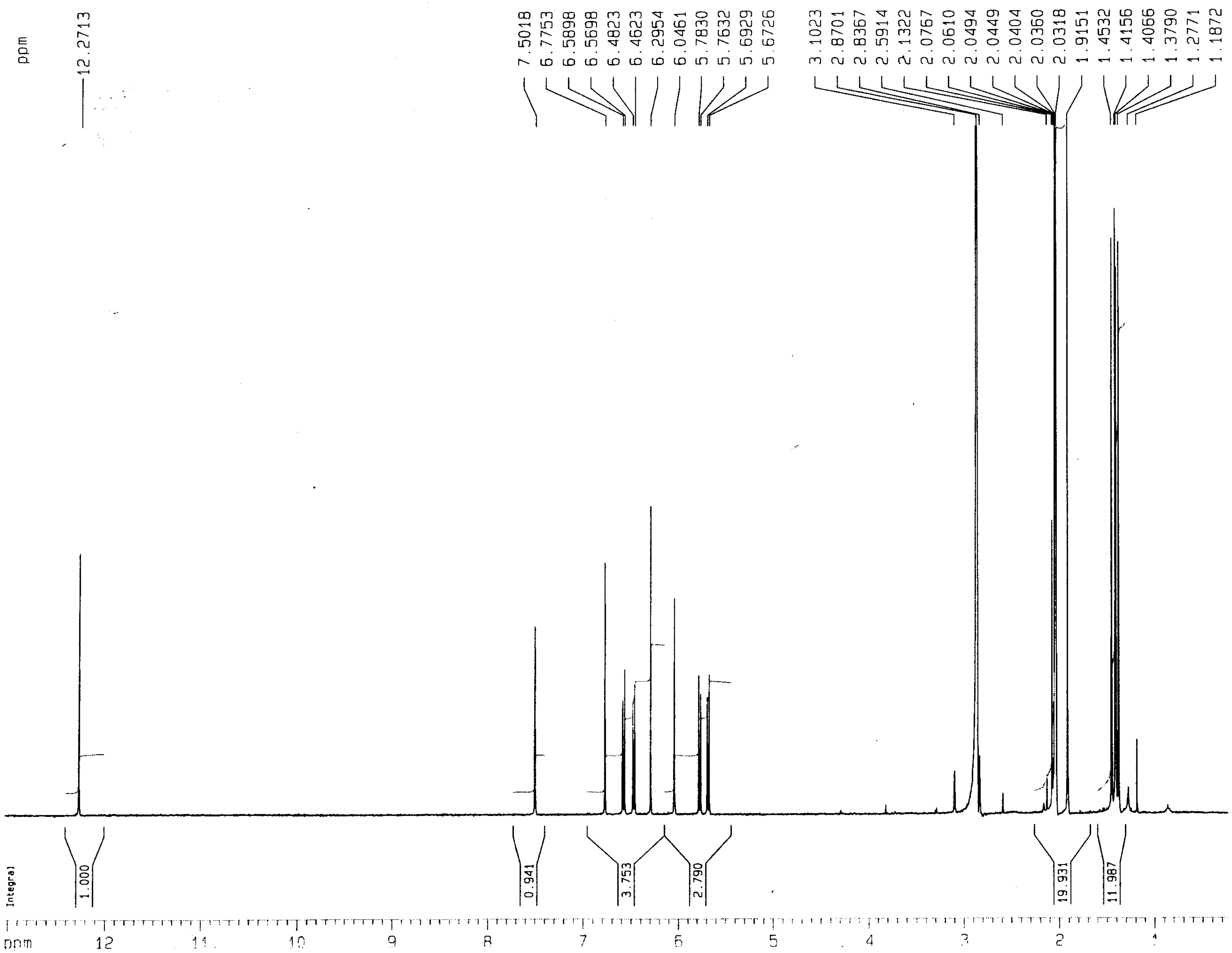

Current Data Parameters

NAME	wtt13
EXPNO	21
PROCNO	1

F2 - Acquisition Parameters

Date_	20080526
Time	17.22
INSTRUM	spect
PROBHD	5 mm DUL 13C-1
PULPROG	zg
TD	32768
SOLVENT	MeOD
NS	1
DS	0
SWH	11467.890 Hz
FIDRES	0.349972 Hz
AQ	1.4287348 sec
RG	362
DW	43.600 usec
DE	6.00 usec
TE	0.0 K
D1	1.00000000 sec
MCREST	0.00000000 sec
MCWRK	0.01500000 sec

======== CHANNEL f1 ========

NUC1	1H
P1	10.00 usec
PL1	-4.00 dB
SFO1	500.0324562 MHz

F2 - Processing parameters

SI	16384
SF	500.0306424 MHz
WDW	GM
SSB	0
LB	-0.40 Hz
GB	0.2
PC	1.00

1D NMR plot parameters

CX	22.00 cm
CY	240.00 cm
F1P	13.037 ppm
F1	6519.06 Hz
F2P	0.237 ppm
F2	118.38 Hz
PPMCM	0.58184 ppm/cm
HZCM	290.94012 Hz/cm

wtt13 c13

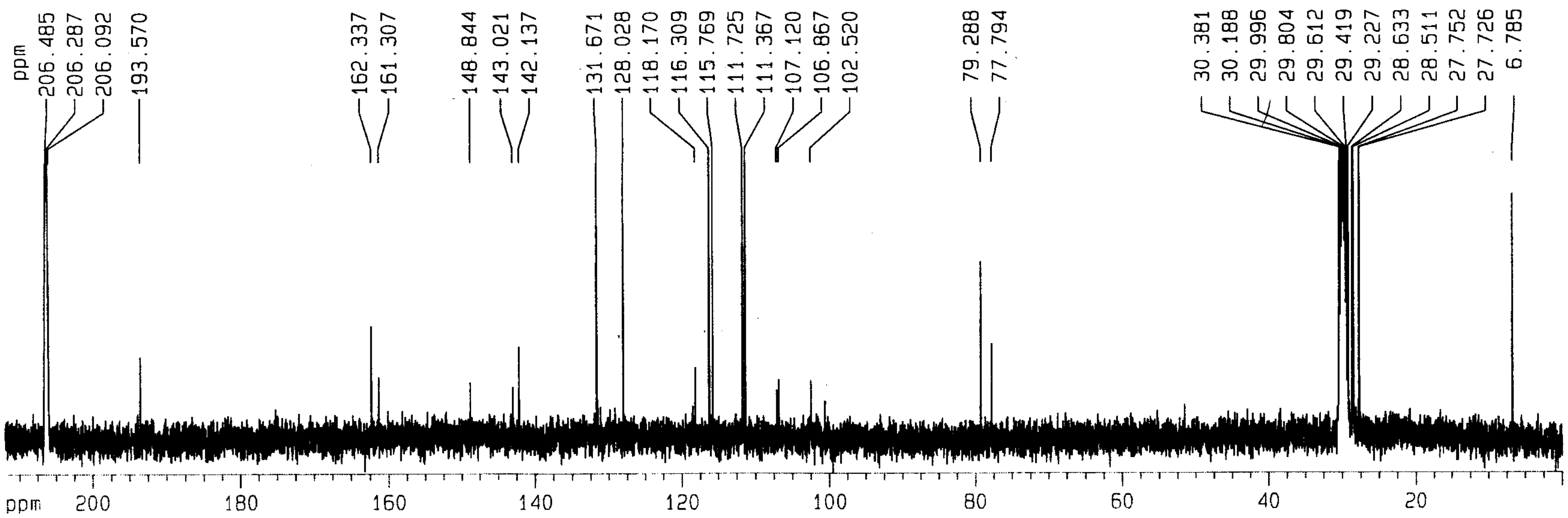

Current Data Parameters

NAME	wtt13
EXPNO	2
PROCNO	1

F2 - Acquisition Parameters

Date_	20080602
Time	7.10
INSTRUM	av400
PROBHD	5 mm QNP 1H/15
PULPROG	zgdc
TD	32768
SOLVENT	Acetone
NS	3400
DS	2
SWH	23584.906 Hz
FIDRES	0.719754 Hz
AQ	0.6947528 sec
RG	71.8
DW	21.200 usec
DE	6.00 usec
TE	291.8 K
D1	3.00000000 sec
d11	0.03000000 sec
MCREST	0.00000000 sec
MCWRK	0.01500000 sec

======== CHANNEL f1 ========

NUC1	13C
P1	9.40 usec
PL1	-4.00 dB
SFO1	100.6239976 MHz

======== CHANNEL f2 ========

CPDPRG2	waltz16
NUC2	1H
PCPD2	90.00 usec
PL2	-3.00 dB
PL12	14.00 dB
SFO2	400.1316005 MHz

F2 - Processing parameters

SI	32768
SF	100.6126855 MHz
WDW	EM
SSB	0
LB	1.00 Hz
GB	0
PC	1.50

1D NMR plot parameters

CX	22.00 cm
CY	6.00 cm
F1P	212.000 ppm
F1	21329.89 Hz
F2P	0.000 ppm
F2	0.00 Hz
PPMCM	9.63636 ppm/cm
HZCM	969.54041 Hz/cm

wtt13 hsqc

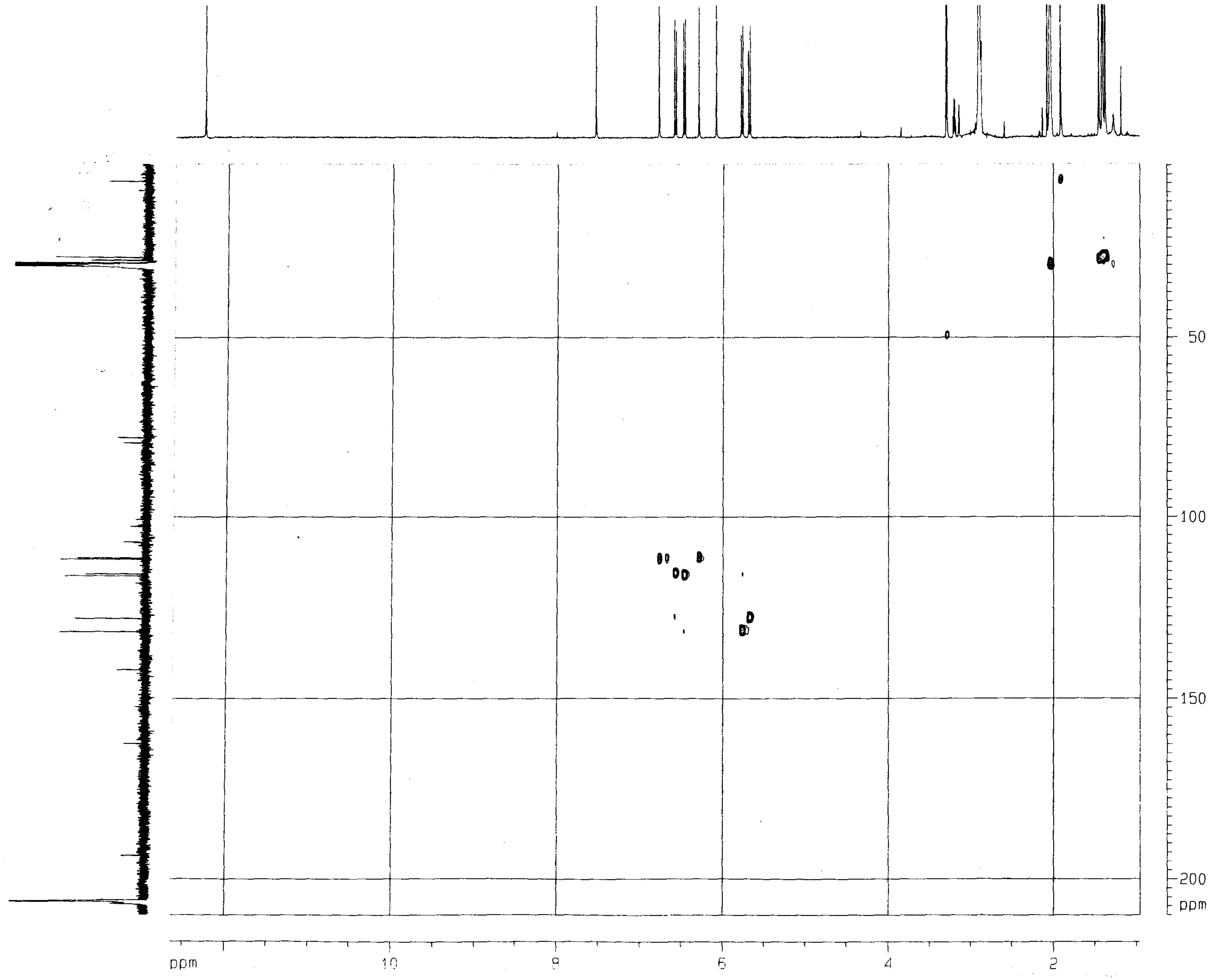

Current Data Parameters

NAME	wtt13	
EXPNO	26	
PROCNO	1	

F2 - Acquisition Parameters

Date_	20080624	
Time	19.58	
INSTRUM	spect	
PROBHD	5 mm BBI 1H-BB	
PULPROG	hsqcetgpsi2	
TD	1024	
SOLVENT	CDCl3	
NS	8	
DS	16	
SWH	6510.417	Hz
FIDRES	6.357829	Hz
AQ	0.0786932	sec
RG	16384	
DW	76.800	usec
DE	6.00	usec
TE	0.0	K
CNST2	145.0000000	
d0	0.00000300	sec
D1	1.50000000	sec
d4	0.00172414	sec
d11	0.03000000	sec
d13	0.00000400	sec
D16	0.00020000	sec
D24	0.00086207	sec
DELTA	0.00127440	sec
DELTA1	0.00120800	sec
DELTA2	0.00006207	sec
DELTA3	0.00052414	sec
IN0	0.00001807	sec
MCREST	0.00000000	sec
MCWRK	0.25000051	sec
ST1CNT	128	

======== CHANNEL f1 ========

NUC1	1H	
P1	9.20	usec
p2	18.40	usec
P28	2000.00	usec
PL1	-1.00	dB
SFO1	500.0339902	MHz

======== CHANNEL f2 ========

CPDPRG2	garp	
NUC2	13C	
P3	12.00	usec
p4	24.00	usec
PCPD2	75.00	usec
PL2	-1.00	dB
PL12	15.50	dB
SFO2	125.7464746	MHz

====== GRADIENT CHANNEL =====

GPNAM1	SINE.100	
GPNAM2	SINE.100	
GPNAM3	SINE.100	
GPNAM4	SINE.100	
GPX1	0.00	%
GPX2	0.00	%
GPX3	0.00	%
GPX4	0.00	%
GPY1	0.00	%
GPY2	0.00	%
GPY3	0.00	%
GPY4	0.00	%
GPZ1	80.00	%
GPZ2	20.10	%
GPZ3	11.00	%
GPZ4	-5.00	%
P16	1000.00	usec
P19	600.00	usec

F1 - Acquisition parameters

ND0	2	
TD	128	
SFO1	125.7425	MHz
FIDRES	216.113419	Hz
SW	219.993	ppm
FnMODE	Echo-Antiecho	

F2 - Processing parameters

SI	1024	
SF	500.0306424	MHz
WDW	QSINE	
SSB	2	
LB	0.00	Hz
GB	0	
PC	1.40	

F1 - Processing parameters

SI	1024	
MC2	echo-antiecho	
SF	125.7287143	MHz
WDW	QSINE	
SSB	2	
LB	0.00	Hz
GB	0	

2D NMR plot parameters

CX2	18.00	cm
CX1	14.00	cm
F2PLO	12.633	ppm
F2LO	6316.95	Hz
F2PHI	0.961	ppm
F2HI	480.46	Hz
F1PLO	210.028	ppm
F1LO	26406.58	Hz
F1PHI	2.050	ppm
F1HI	257.72	Hz
F2PPMCM	0.64846	ppm/cm
F2HZCM	324.24927	Hz/cm
F1PPMCM	[illegible]	ppm/cm
F1HZCM	[illegible]	Hz/cm

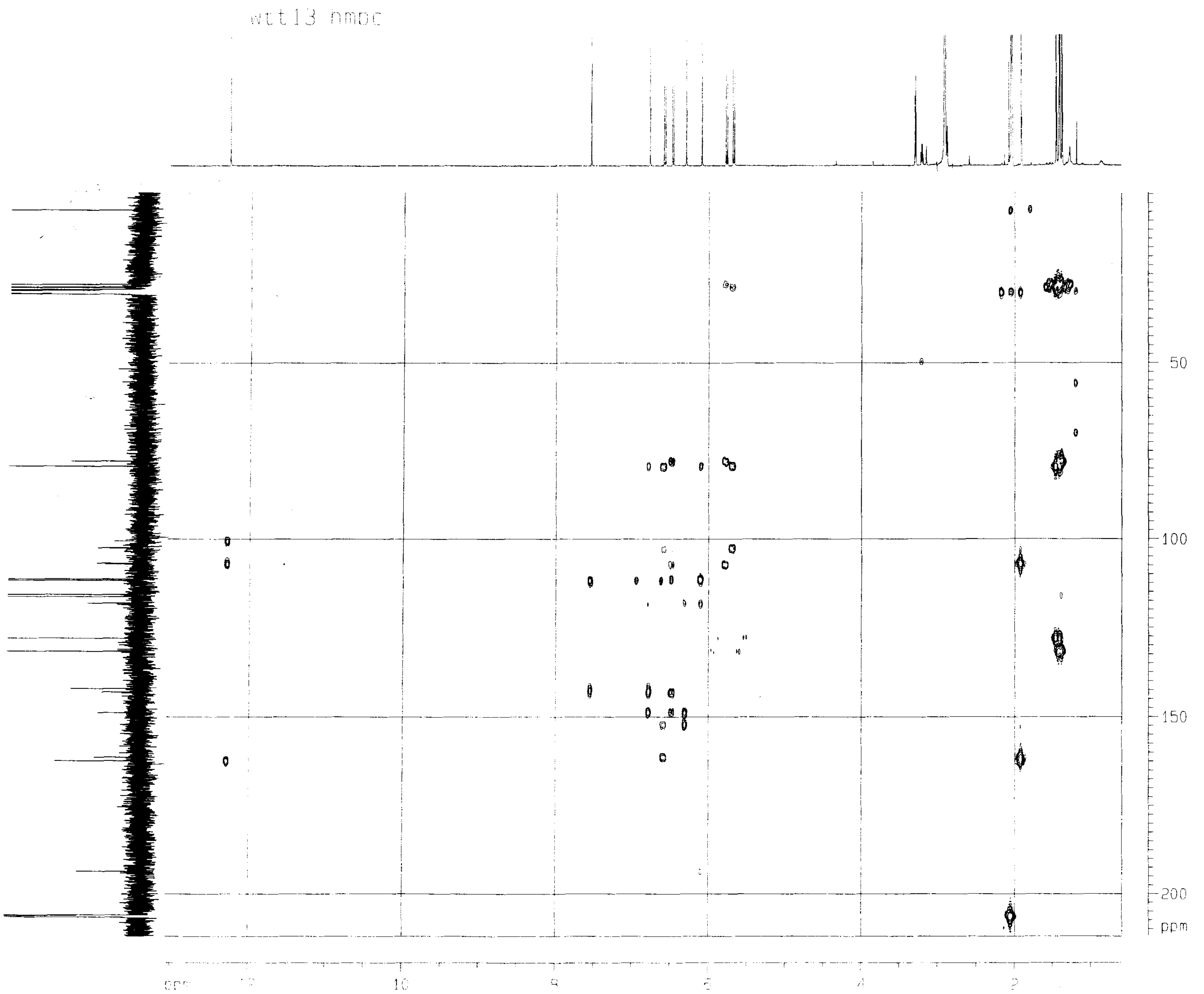

Current Data Parameters

NAME	wtt12
EXPNO	65
PROCNO	1

F2 - Acquisition Parameters

Date_	20080625
Time	21.24
INSTRUM	spect
PROBHD	5 mm BBI 1H-BB
PULPROG	inv4gplplrndqf
TD	2048
SOLVENT	Acetone
NS	78
DS	16
SWH	8992.806 Hz
FIDRES	4.391018 Hz
AQ	0.1139188 sec
RG	18390.4
DW	55.600 usec
DE	6.00 usec
TE	0.0 K
CNST12	145.0000000
d0	0.00000300 sec
D1	1.39999998 sec
d2	0.00344828 sec
D6	0.08340000 sec
d13	0.00000400 sec
D16	0.00020000 sec
IN0	0.00001791 sec
MCREST	0.00000000 sec
MCWRK	1.39999998 sec

======== CHANNEL f1 ========

NUC1	1H
P1	9.20 usec
p2	18.40 usec
PL1	-1.00 dB
SFO1	500.0335002 MHz

======== CHANNEL f2 ========

NUC2	13C
P3	12.00 usec
PL2	-1.00 dB
SFO2	125.7466003 MHz

====== GRADIENT CHANNEL =====

GPNAM1	SINE.100
GPNAM2	SINE.100
GPNAM3	SINE.100
GPX1	0.00 %
GPX2	0.00 %
GPX3	0.00 %
GPY1	0.00 %
GPY2	0.00 %
GPY3	0.00 %
GPZ1	50.00 %
GPZ2	30.00 %
GPZ3	40.10 %
P16	1000.00 usec

F1 - Acquisition parameters

ND0	2
TD	128
SFO1	125.7466 MHz
FIDRES	218.073975 Hz
SW	221.982 ppm
FnMODE	QF

F2 - Processing parameters

SI	1024
SF	500.0300058 MHz
WDW	SINE
SSB	0
LB	0.00 Hz
GB	0
PC	1.40

F1 - Processing parameters

SI	512
MC2	QF
SF	125.7325281 MHz
WDW	SINE
SSB	0
LB	0.00 Hz
GB	0

2D NMR plot parameters

CX2	18.00 cm
CX1	14.00 cm
F2PLO	13.065 ppm
F2LO	6533.04 Hz
F2PHI	0.596 ppm
F2HI	297.80 Hz
F1PLO	212.085 ppm
F1LO	26666.02 Hz
F1PHI	1.786 ppm
F1HI	224.57 Hz
F2PPMCM	0.69275 ppm/cm
F2HZCM	[illegible]
[illegible]	[illegible]
[illegible]	[illegible]

C:\Xcalibur\data\080602e-07 06/02/2008 05:03:54 PM wtt-13

080602e-07 #40-45 RT: 1.75-1.91 AV: 6 SB: 2 2.07, 2.07 NL: 8.74E6
T: + c Full ms [50.00-1000.00]

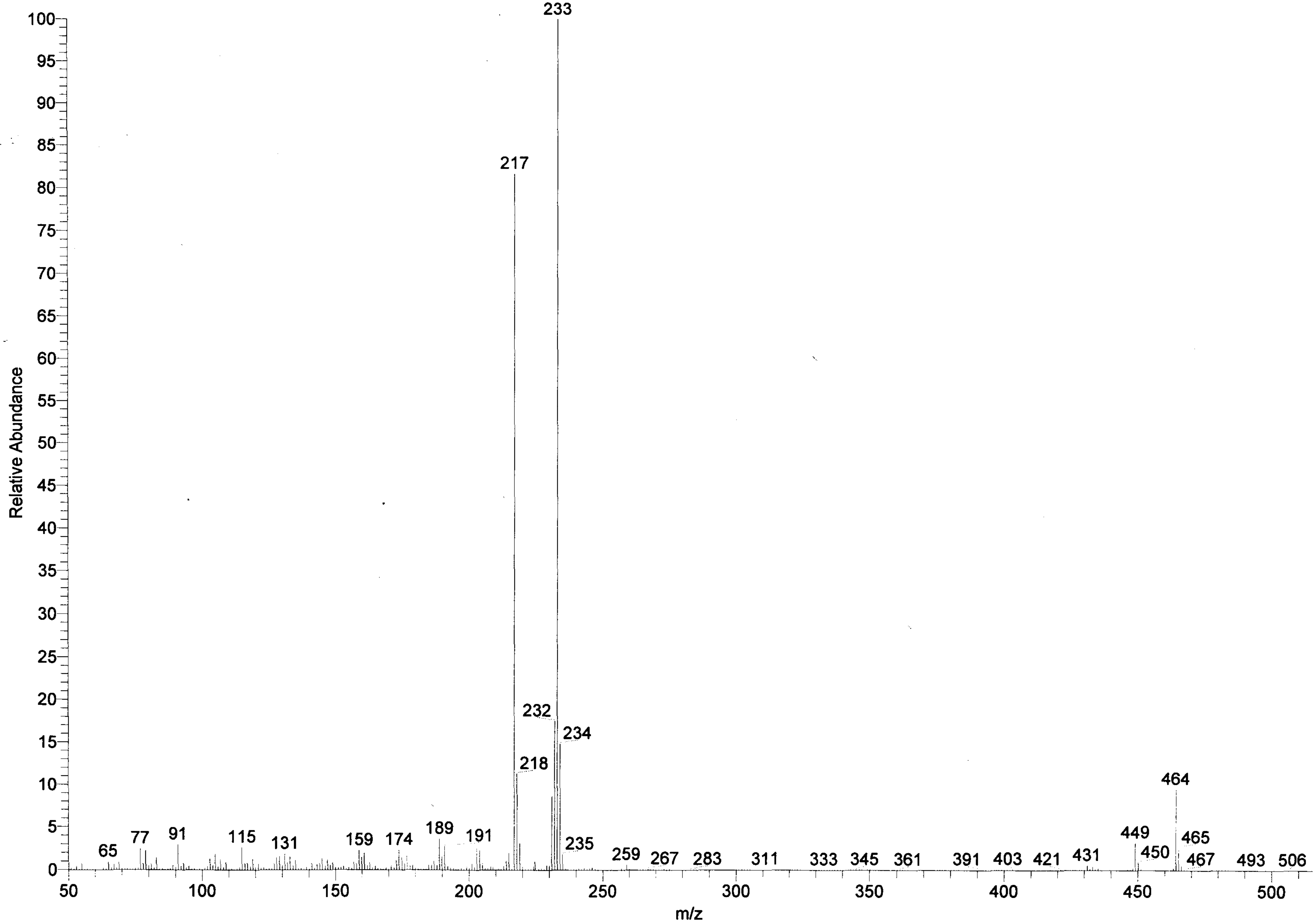

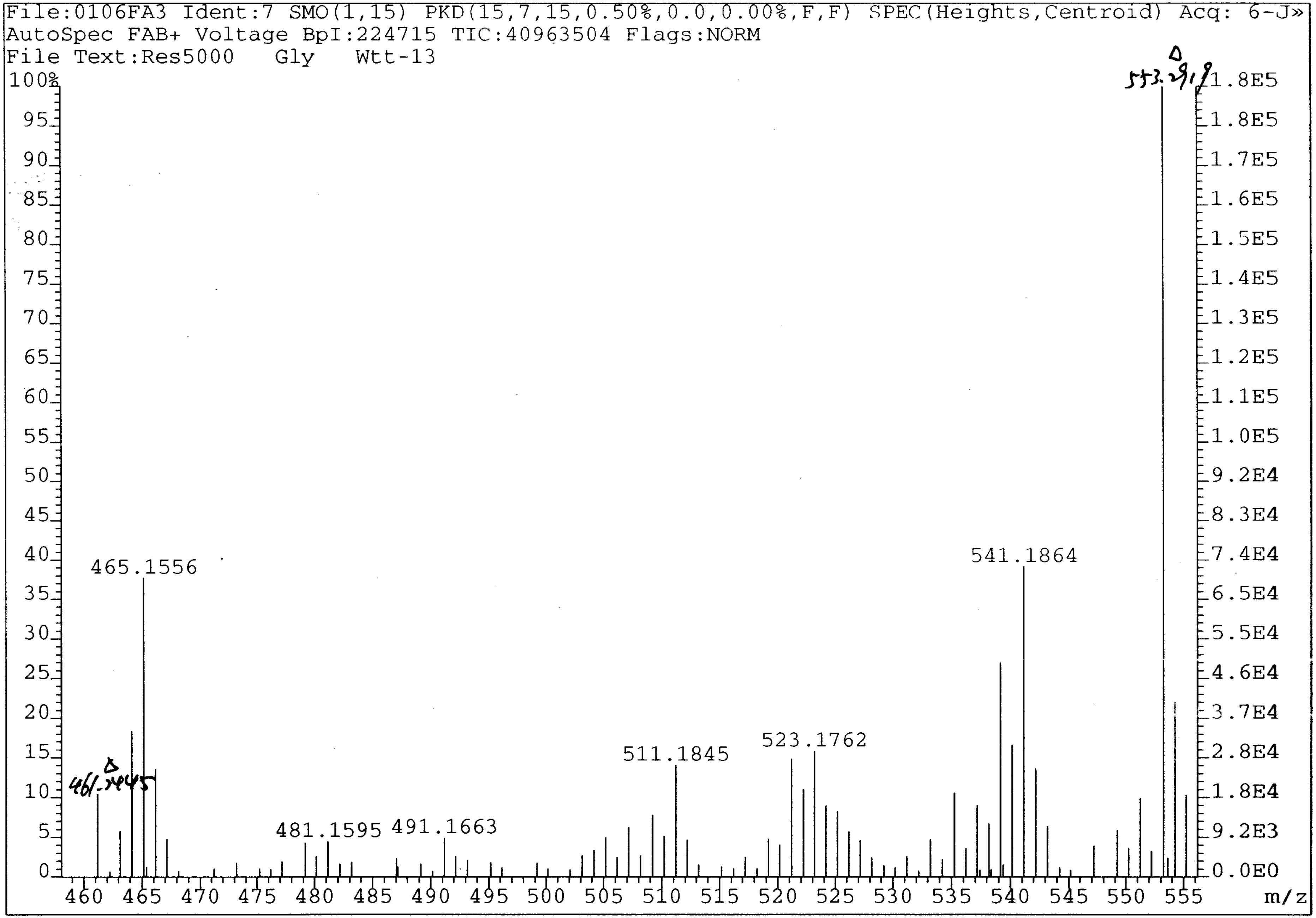

File:0106FA3 Ident:7 SMO(1,15) PKD(15,7,15,0.50%,0.0,0.00%,F,F) SPEC(Heights,Centroid) Acq: 6-J»
AutoSpec FAB+ Voltage BpI:224715 TIC:40963504 Flags:NORM
File Text:Res5000 Gly Wtt-13
465.1556
481.1595
491.1663
511.1845
523.1762
541.1864
m/z

File:0106FA3 Ident:7 SMO(1,15) PKD(15,7,15,0.50%,0.0,0.00%,F,F)
AutoSpec FAB+ Voltage BpI:224715 TIC:40963504 Flags:NORM
File Text:Res5000 Gly Wtt-13
Heteroatom Max: 60 Ion: Both Even and Odd
Limits:

				-0.5	0	0	0
465.155649	10.0			20.0	200	400	10

Mass	mDa	PPM	Calc. Mass	DBE	C	H	O
465.155649	-0.7	-1.5	465.154943	14.5	26	25	8

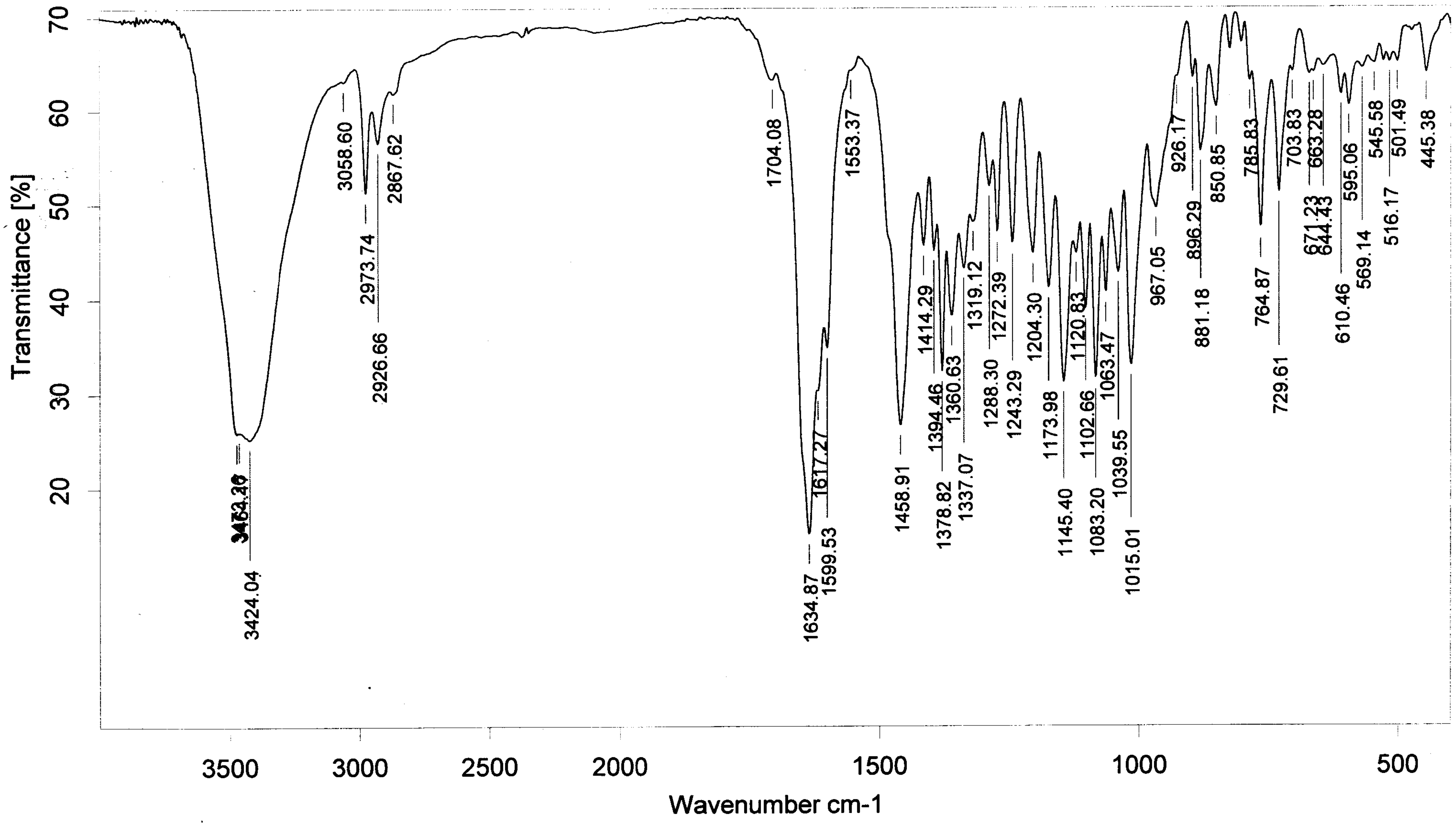

Sample : Wtt-13	Frequency Range : 399.271 - 3996.57	Measured on : 30/12/2008	
Technique : KBr压片	Resolution : 4	Instrument : Tensor27	Sample Scans : 16
Customer : 081230IR3	Zerofilling : 2	Acquisition : Double Sided,For	

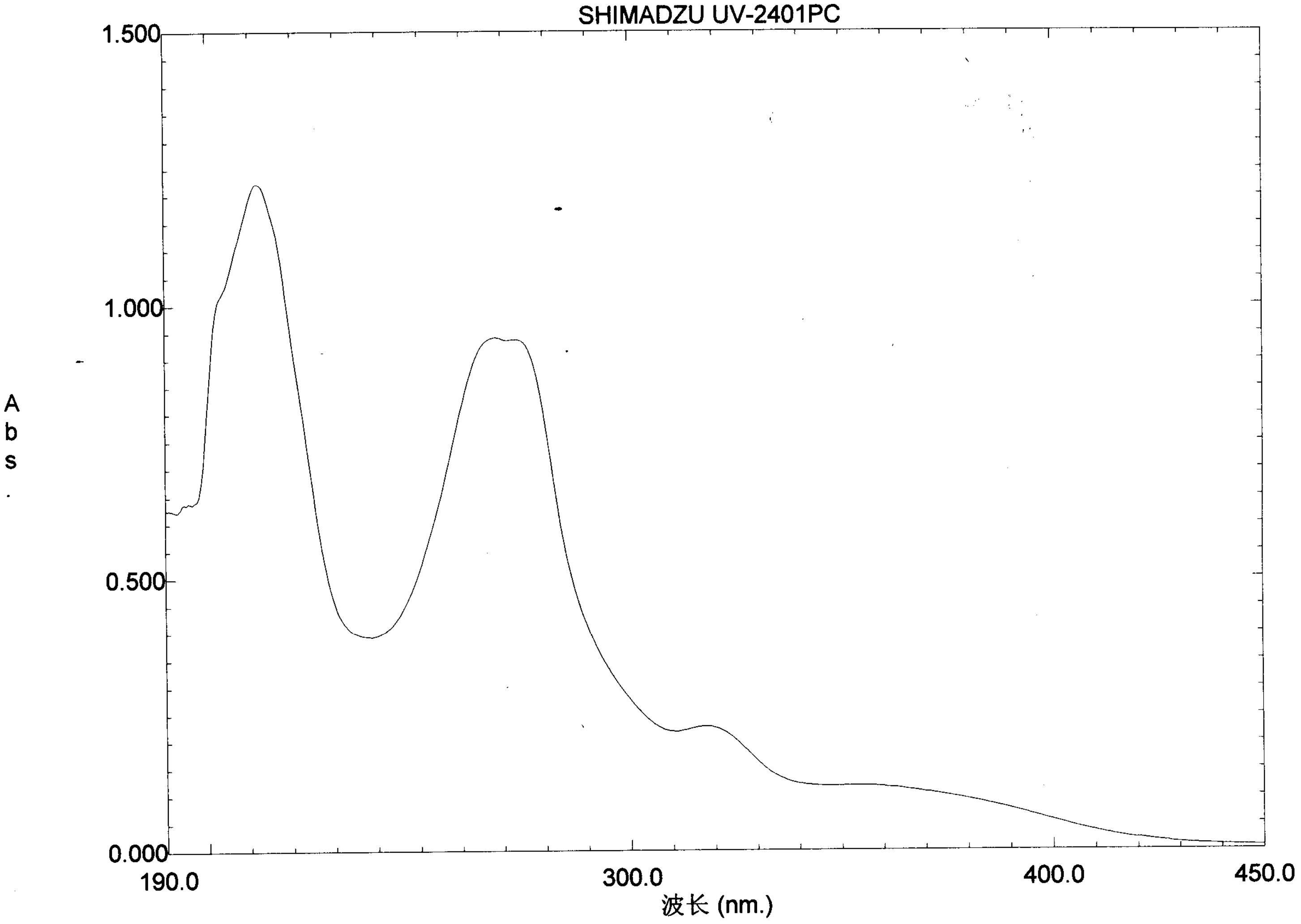

文件名: WTT-13

WTT-13 ———

样品浓度：0.010毫克/毫升
溶剂：甲醇

创建于: 14:59 08-12-25
数据: 原始

测量模式: Abs.
扫描速度: 中速
狭缝: 2.0
采样间隔: 0.5

否.	波长 (nm.)	Abs.
1	875.50	-0.0020
2	355.50	0.1176
3	318.50	0.2267
4	273.50	0.9367
5	268.50	0.9402
6	212.00	1.2226
